# Purification, characterization and three-dimensional structure prediction of multicopper oxidase Laccases from *Trichoderma lixii* FLU1 and *Talaromyces pinophilus* FLU12

**DOI:** 10.1038/s41598-024-63959-z

**Published:** 2024-06-11

**Authors:** Samson O. Egbewale, Ajit Kumar, Mduduzi P. Mokoena, Ademola O. Olaniran

**Affiliations:** 1 Discipline of Microbiology, School of Life Sciences, College of Agriculture, Engineering and Science, University of KwaZulu-Natal (Westville Campus), Durban, 4001 South Africa; 2 Department of Pathology, School of Medicine, University of Limpopo, Private Bag X1106, Sovenga, 0727 South Africa

**Keywords:** *Trichoderma lixii*, *Talaromyces pinophilus*, Laccases, Polycyclic aromatic hydrocarbons (PAHs), Biodegradation, Biochemistry, Biotechnology, Computational biology and bioinformatics, Microbiology, Environmental sciences

## Abstract

Broad-spectrum biocatalysts enzymes, Laccases, have been implicated in the complete degradation of harmful pollutants into less-toxic compounds. In this study, two extracellularly produced Laccases were purified to homogeneity from two different Ascomycetes spp. *Trichoderma lixii* FLU1 (*Tl*FLU1) and *Talaromyces pinophilus* FLU12 (*Tp*FLU12). The purified enzymes are monomeric units, with a molecular mass of 44 kDa and 68.7 kDa for *Tl*FLU1 and *Tp*FLU12*,* respectively, on SDS-PAGE and zymogram. It reveals distinct properties beyond classic protein absorption at 270–280 nm, with *Tl*FLU1's peak at 270 nm aligning with this typical range of type II Cu site (white Laccase), while *Tp*FLU12's unique 600 nm peak signifies a type I Cu^*2*+^ site (blue Laccase), highlighting the diverse spectral fingerprints within the Laccase family. The *K*_m_ and *k*_cat_ values revealed that ABTS is the most suitable substrate as compared to 2,6-dimethoxyphenol, caffeic acid and guaiacol for both Laccases. The bioinformatics analysis revealed critical His, Ile, and Arg residues for copper binding at active sites, deviating from the traditional two His and a Cys motif in some Laccases. The predicted biological functions of the Laccases include oxidation–reduction, lignin metabolism, cellular metal ion homeostasis, phenylpropanoid catabolism, aromatic compound metabolism, cellulose metabolism, and biological adhesion. Additionally, investigation of degradation of polycyclic aromatic hydrocarbons (PAHs) by purified Laccases show significant reductions in residual concentrations of fluoranthene and anthracene after a 96-h incubation period. *Tl*FLU1 Laccase achieved 39.0% and 44.9% transformation of fluoranthene and anthracene, respectively, while *Tp*FLU12 Laccase achieved 47.2% and 50.0% transformation, respectively. The enzyme structure–function relationship study provided insights into the catalytic mechanism of these Laccases for possible biotechnological and industrial applications.

## Introduction

﻿Fungi belonging to the phylum Ascomycota are well known for rapid growth, utilization of diverse substrate ranges, and degradation of the xenobiotics^1^. Among these fungi, the families *Hypocreaceae* and *Trichocomacea* are not only studied primarily for their role as biological control agents against numerous pathogens^2,3^, but also for their versatile enzymatic system including Laccases, Peroxidases and Oxidases which are crucial for degrading lignin and other recalcitrant aromatic compounds of environmental concerns^4^. Among these groups of versatile extracellular enzymes, Laccases have continued to receive significant attention in the past decade because of high redox potentials, its metal tolerance properties^5,6^ and industrial applications, such as polyaromatic hydrocarbons (PAHs) bioremediation, delignification or second-generation ethanol production, pulp bleaching, dye decolorization and detoxification of xenobiotics^7–10^.

Laccase (Benzenediol oxygen oxidoreductases, EC 1.10.3.2) belongs to the multi-copper class of enzymes with the potential of oxidizing aromatic and non-aromatic compounds to an innocuous state﻿, unlike other ligninolytic enzymes such as Manganese peroxidase (MnP) and Lignin peroxidase (LiP), which require hydrogen peroxide for activity^11^. Laccases ubiquitously found in fungi, higher plants, some insects, and a few bacteria^12^. The production of these enzymes is always triggered in response to environmental stress and nutrient depletion^7^. The physiological function of Laccase varies depending on the organisms; in plants these enzymes are involved in the lignification processes^13^, in insects, it aids the formation of cuticles during the sclerotization process^14^, in fungi, it helps the morphogenesis process (formation of spores, pigments of fruiting bodies)^15,16^, pathogenesis^17^, virulence^18^, lignin degradation, monomer cross-linking, polymer degradation^19^, and also the breakdown of aromatic rings^19^. The precise role of Laccase in bacteria remains unclear but few studies have shown its functions in industrial applications requiring activity at high pH and temperatures as robust biocatalysts^20^. Fungal Laccases are glycoproteins which exist as monomer, homodimer, heterodimer, and multimer forms, with four redox-active copper atoms used in oxidizing substrates using oxygen as electron acceptor and its subsequent reduction to form water molecule^21^. The molecular mass and isoelectric point (pl) of Laccases ranges from 50 to 100 kDa and 3–7, respectively^22^. Typical Laccase consists of three Cu centres (Type I, II and III) with UV–vis spectrum absorption between 280 to 610 nm and a shouldering at 330 nm^23^. Type I Cu is responsible for the intense blue colour with UV–vis absorption spectrum at 605 nm and detectable in electro paramagnetic resonance (EPR) spectrum with narrow hyperfine coupling characteristics due to an S-Cu LMCT transition (ligand-to-metal charge transfer)^24,25^. Type II, unlike type I, is colourless and EPR detectable, while Type III Cu, on the other hand, has a pair of Cu atoms which have weak absorbance in the UV–vis spectrum and lacks an EPR spectrum^24^. Both. Type II and III Cu, forms the trinuclear cluster of Laccases where dioxygen binds and four-electron reduction to water takes place^26^. Different fungal species with blue Laccase production characteristics and PAHs degradation potential includes *Trametes versicolor*^27^, *Trichoderma viride* (EXF8977), *Penicillium chrysogenum* (EXF1818) and *Irpex lacteus* (MUM 04.98)^28^, while the use of a monomeric yellow Laccase in *Leucoagaricus gongylophorus* (Lac1Lg) has been documented in the oxidization of anthracene^29^.

Despite the wide distribution and importance of Laccases, detailed studies on different Laccases in ascomycetes and their potential application in transforming organo-pollutants like PAHs into an innocuous state are scanty. Additionally, systematic spectral studies and bioinformatic analyses of ascomycete Laccases remain limited. Hence, this study aims to fill these knowledge gaps and contribute to the development of more effective strategies for PAH bioremediation using ascomycete Laccases by purifying and characterizing from *Trichoderma lixii* FLU1 and *Talaromyces pinophilus* FLU12, two ascomycetes isolated from benzo(b)fluoranthene activated sludge and known for degrading anthracene and fluoranthene. Furthermore, the substrate binding mechanism, homology modelling and prediction of their three-dimensional structure were determined.

## Materials and methods

### Reagents

ABTS (2,2-azino-bis-(3-ethyl-benzothiazoline-6-sulphonic acid)), Basal Salt Medium (BSM), anthracene, benzo (b) fluoranthene (BbF), 2,6-dimethoxyphenol (DMP), (NH_4_)_2_SO4 DEAE-cellulose and Sephadex G-100 were purchased from Merck (Burlington, MA, USA). All other chemicals used were of the highest purity and analytical grade.

### Fungal strains and molecular identification

The isolation and identification of the two fungal strains, *Trichoderma lixii* FLU1 (*Tl*FLU1) and *Talaromyces pinophilus* FLU12 (*Tp*FLU12), used in this study have been reported previously^30^. Briefly, the strains were isolated from benzo(b)fluoranthene-enriched activated sludge which was later serially diluted and plated on potato dextrose agar (PDA) medium containing 100 mg/L of benzo(b)fluoranthene. The plates were incubated at 30 °C for 7 days and observed for the appearance of fungal colonies. The colonies that showed clear zones were selected as potential benzo(b)fluoranthene-degrading fungi, purified by repeated subculturing on fresh PDA plates and maintained on PDA slants at 4 °C. Due to the hydrophobic nature of benzo(b)fluoranthene, a superficial concentration of 100 mg/L was achieved on a PDA agar plate by evenly spreading it across the surface using a sterile glass rod and then left in the laminar hood for 2 h to allow the solvent used to prepare benzo(b)fluoranthene to evaporate which ensures a strict adherence of benzo(b)fluoranthene to the agar surface.

Later, the purified fungal strains were identified by morphological and molecular methods. The morphological characteristics of the fungal strains were examined by light microscopy and compared with standard references. The molecular identification was performed by extracting the genomic DNA from the fungal strains and amplifying the internal transcribed spacer (ITS) region of the ribosomal RNA gene by PCR. The PCR products were sequenced and compared with the sequences available in the GenBank database. The phylogenetic analysis was conducted by using the MEGA software.

### Culture conditions for Laccase production

Each fungal strain was separately cultured in cotton-plugged Erlenmeyer flasks (250 mL) containing BSM and anthracene (200 mg/L) as an inducer to a final volume of 150 mL prior to the set-up. The media were sterilized by autoclaving at 121 °C for 15 min. Inoculation was done directly into individual Erlenmeyer flasks using two 20 mm mycelial disks of each strain before incubating at 30℃ and shaking at 180 rpm for 10 days in complete darkness (MRC laboratory instrument, Essex, U.K). The pH of the flask containing strain *Tl*FLU1 was adjusted to 4 using 1 M HCl while that of *Tp*FLU12 was adjusted to 7 using 1 M NaOH. The details of the specific components and concentration for the BSM media and PDA are presented in Table S1. The variation in the culture media pH was observed due to optimized condition during anthracene degradation with 100% degradation efficiency after 12 days.

### Purification of Laccase

The purification was carried out according to the previously described method (Othman et al., 2018) with some modifications. Briefly, crude extract from *Tl*FLU1 and *Tp*FLU12 was individually centrifuged at 5,000 × *g* for 20 min at 4℃ to obtain a clear supernatant. Protein from each supernatant was sequentially precipitated to saturation with 60% (NH_4_)_2_SO_4_ under a gentle continuous stirring at 4℃ overnight. The protein pellets were recovered by centrifugation at 10,000 × *g* for 20 min, dissolved in sodium acetate buffer (50 mM, pH 5) and dialyzed against a large volume of the same buffer using 30 kDa cut off size dialysis tubing cellulose membrane (Merck, Burlington, MA, USA). The protein was further purified using DEAE liquid chromatography column (1.5 × 9 cm) at room temperature before eluting with 100 mM NaCl in 50 mM sodium acetate buffer (pH 7) at a flow rate of 0.5 ml/min. ﻿Six fractions showing Laccase enzymes activity of substrate ABTS were pooled and applied on ﻿Sephadex G-100 column (2.0 × 9 cm) size exclusion column before elution with 50 mM sodium acetate buffer (pH 7). ﻿All fractions with Laccase activity were pooled, desalted, filter-sterilized, and stored at 4℃ until further usage.

### Protein quantification and enzyme activity

The protein concentrations were determined as described previously^31^ using bovine serum albumin (BSA) as standard. Laccase activity was determined according to previously described method^32^ with slight modifications. Briefly, the reaction mixture contained 100 μL of 50 mM ABTS and 800 μL of 20 mM sodium acetate buffer (pH 5) and an appropriately diluted purified enzyme (370 μg). The mixture was incubated at 30℃ for 15 min and the reaction was terminated with 40 μL of 20% trichloroacetic acid. One unit of Laccase is the amount of enzyme that produces 1 µM of oxidized product per minute (ε_420 nm_ = 36,000 M^−1^ cm^−1^ for oxidized product).

### Zymogram analysis

Non-denaturing polyacrylamide gel electrophoreses (﻿Zymogram) were used to confirm the protein purity and activity^33^. The sample loading buffer was prepared without the addition of β-mercaptoethanol (non-reducing condition), and the samples (100 µg for *Tl*FLU1 and 75 µg for *Tp*FLU12 of total protein) were not heated before loading into the wells. To detect the Laccase activity after the electrophoresis, the gel was washed once with a mixture of equal parts of isopropanol-sodium acetate buffer (50 mM, pH 5.0) for 30 min and once with sodium acetate buffer for 30 min, to remove the SDS, fix the proteins in the gel, and to reduce the pH to 5.0. The gel was then transferred onto a glass plate before layering with ABTS-agar (20 mg of ABTS, 400 mg of agar, 40 mL of water; heated to dissolve agar). These layers were incubated at 25 °C until the appearance of green bands.

### UV–visible absorption spectrum and FTIR analysis

To study the copper active centre of purified enzymes, 1 mL of enzymes (370 µg of total protein) was added to a 10 mm optical path quartz cuvette and absorbance was measured in a scanning mode wavelength from 200–800 nm using Agilent Cary 60 UV–Vis spectrophotometer (Agilent, Santa Clara, CA, USA)^34^. To monitor functional groups of the purified proteins (370 µg of total protein), FTIR analysis was performed as described previously^30^.

### Catalytic properties of the purified Laccases

﻿ Laccase activity was assayed by using several substrates. Assays were performed in 50 mM of the appropriate buffer and pH, and the oxidized products were read at the appropriate wavelength, as follows: 5 mM ABTS (ε_420_ = 36.00 cm^-1^ mM^−1^), 5 mM 2,6-DMP (_ε469_ = 49.60 cm^−1^ mM^−1^), 5 mM guaiacol (_ε465_ = 12.10 cm^−1^ mM^−1^) and 5 mM caffeic acid (_ε312_ = 11.20 cm^−1^ mM^−1^). The optimum pH for Laccase activity was investigated by using purified enzyme prepared in a reaction mixture containing the appropriate substrate in buffers as follows: 50 mM citrate buffer pH 1–6, 50 mM acetate buffer pH 3–5, 50 mM phosphate buffer pH 6–8 and 50 mM Tris buffer at pH 7 and 11. The optimum temperature of Laccase activity was investigated by using each purified enzyme (370 µg of total protein) for *Tl*FLU1 and *Tp*FLU12, respectively) prepared separately in a reaction mixture containing 5 mM ABTS in 50 mM acetate buffer pH 5, which was incubated at different temperatures (5, 10, 20, 30, 40, 50, 60, 70, 80, 90 and 100 °C) for 1 h. Also, the pH stability and thermal stability of the purified Laccase were studied by determining the residual activity for each enzyme (370 µg of total protein) after 24 h incubation under the same assay conditions for the optimum pH and temperature in darkness using ABTS as substrate. The reaction was monitored at 420 nm using Agilent Cary 60 UV–Vis spectrophotometer (Agilent, Santa Clara, CA, USA). The effect of appropriate substrate concentration (ABTS, 2,6-DMP, guaiacol and caffeic acid) on initial reaction velocity catalysed by purified Laccase was also investigated at constant enzyme concentration (370 µg of total protein). Therefore, as described above, the purified enzyme was assayed using varying concentrations (1–5 mM) of each substrate in 50 mM acetate buffer pH 5 and 7 at 30 and 50 °C for Laccase from *Tl*FLU1 and *Tp*FLU12, respectively. Michaelis–Menten reciprocal plots were used to determine the kinetic constants (*v*_max_, *K*_m,_ *k*_cat_ and *k*_cat/_*K*_m_) for appropriate substrate using the Graph-Pad Prism 8.0 (San Diego, USA) software. The residual enzyme activity at varied concentrations of metal ions (10, 50 and 100 mM: Al^3+^, As^5+^, Ca^2+^, Cd^2+^, Co^2+^, Cu^2+^, Fe^2+^, K^+^, Li^+^, Mg^2+^, Mn^2+^, Mo^+^, Na^+^, Ni^2+^ and Zn^2+^), Organic solvent (10, 50 and 90%: 1,4 Dioxane, acetone, acetonitrile, butanol, DMSO, ethanol, ethyl-acetate, isopropanol, methanol and toluene) and inhibitors (DTT, 0.01 and 0.1 mM; EDTA, 1 and 10 mM; NaN_3_, 0.05 and 0.1 mM, ﻿SDS 5 and 10 mM; Urea, 20, 50 and 100 mM) was investigated by pre-incubating the purified enzymes (370 µg of total protein) for 24 h.

### In-gel trypsin digestion and identification of the purified Laccase in ESMS

Purified Laccase (100 μg) from *Tl*FLU1 and *Tp*FLU12 was loaded onto 10% SDS-PAGE and stained with Coomassie blue R250. Protein bands were excised, digested with trypsin and subjected to electrospray mass spectrometry (ES-MS) for amino acid fragment identification at the Central Analytical Facility of Stellenbosch University, Stellenbosch, South Africa. The raw files generated by the mass spectrometer were imported into Proteome Discoverer v1.4 and processed using the Sequest algorithm against the UniProt (www.uniprot.org) *Trichoderma* genus and *Talaromyces* genus database, limited to Laccases. The peptide sequences were inferred from these matches with validation using the Target-Decoy PSM validator node. The search results were further validated using Scaffold Q + ^35^.

### Template-based structure prediction, homology modelling and Laccase ligand binding site prediction for Laccase

The three-dimensional structure of the protein was predicted by submitting the amino acid sequence to the I-TASSER servers^36^. LOMETS approach was used to search for templates of similar folds from the PDB library^37,38^. Ten templates were used to build homology models while the top model structure was selected to optimize using the PyMOL Molecular Graphics System program (Schrödinger LLC., USA). The model quality validation through the 3D protein structure assessment, Errat and Ramachandran plot was performed using the Schrödinger Maestro software (Schrödinger LLC., NY, USA). Structural homology alignment was constructed with Jalview 2.11.2.4^39^. Additionally, the Laccase ligand binding sites residues, their molecular function, biological process, and cellular components were predicted using I-TASSER servers in accordance with the ancestor chart of the EMBL’s European Bioinformatics institute gene ontology (GO) terms.

### Sequence alignment

Detection of Laccase amino acid sequences from *Tl*FLU1 and *Tp*FLU12 similarities with amino acid sequences of Laccase from *Trichoderma species* and *Talaromyces species* deposited at UniProtKB were analysed using the BLAST tool (http://uniprot.org/blast). A phylogenetic approach was used to determine the taxonomic classification while amino acid sequence alignment was performed using muscle (multiple sequence comparison by UMPGA) as implanted in MEGA 11. Alignments were examined manually and a phylogenetic tree was constructed with a neighbour-joining likelihood approach and bootstrap resampling of 1000 replicates using MEGA 11^40^.

### Degradation of PAHs by Laccase

The two PAHs (fluoranthene and anthracene) were selected based on their designated profile as known or reasonably anticipated human carcinogens on US EPA’s priority pollutants list^41^. The assays were carried out as described previously^42^ with some modifications. Briefly, 5 mL reaction mixture contained anthracene and fluoranthene (200 mg/L) individually in a 15 mL centrifuge tube, Laccase (2 U/ml) and sodium acetate buffer (50 mM, pH 5 and 7 for *Tl*FLU1 and *Tp*FLU12, respectively). Tween 80 (1%) was added to the mixture to enhance PAH bioavailability and shaken continuously at 80 rpm to avoid PAHs precipitation at 30 °C in darkness for 96 h (MRC Lab suspension mixer SM-3600, U.K). The set up was performed in triplicate while samples were drawn every 24 h for residual PAHs quantification. 50 μl of 20% (v/v) TCA solution was constantly added to each draw sample before quantifying residual PAHs to stop further enzymatic oxidation. A control set up was carried out under the same experimental condition except for the Laccase being heated denatured. However, extraction and quantification of the residual PAHs was carried out as previously^30^. The mixture was then vigorously shaken for 5 min and allowed to stand for 20 min to enhance the separation of aqueous and organic phases using the standard extraction method. The organic phase was collected, dried over 10 g anhydrous Na_2_SO_4_ and evaporated to dryness at 40 °C under reduced pressure. The dried fractions were then redissolved with the same extraction solvent and diluted tenfold with ethyl-acetate before quantifying the residual anthracene in a scanning spectrum mode using Agilent Cary 60 UV–Vis spectrophotometer from a wavelength of 200 to 400 nm. The absorption spectrum of anthracene and fluoranthene shows peaks at λ_max_ 286 and 265 nm respectively, using a quartz cuvette with an optical path of 10 mm. However, the reaction medium's anthracene and fluoranthene residual concentrations were determined through extrapolation from a standard curve with an *R*^*2*^ value of 0.972 and 0.958 respectively. Also, a recovery study was carried out in triplicate with a sterile sodium acetate buffer with heated-denatured Laccase to an appropriate volume of known concentrations of each PAH compounds as standard, under static conditions and subjected to PAH determination with an average recovery of 98.7 ± 0.53% and 97.9 ± 7.17%, respectively, for anthracene and fluoranthene.

### Statistics analysis

All data were collected in triplicates. Data were statistically analysed using the Graph-Pad Prism 8.0 (San Diego, USA) software and expressed as the means ± standard error. For determining *K*_m_ and *v*_max_, the Michaelis–Menten equation was fitted directly to the experimental data using the nonlinear least-squares fitting procedure of the Graph-Pad Prism 8.0 software.

## Results

### Purification of Laccases

**Figure 1:**
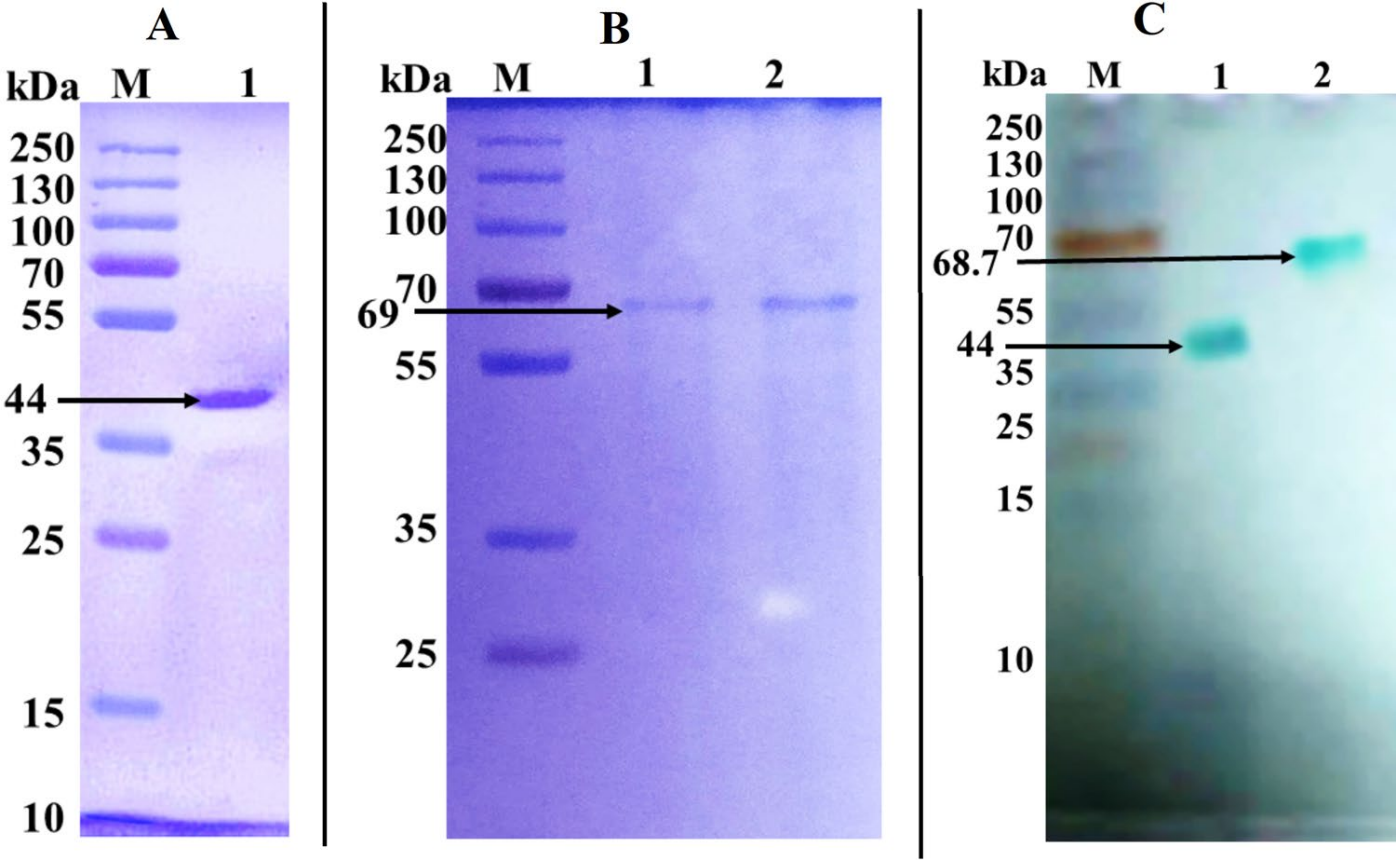
SDS-PAGE and native page showing single bands and in gel activity of Laccases. (**A**) Lane M: protein marker, Lane 1: purified Laccase from *Tl*FLU1; (**B**) Lane M: protein marker, Lane 1–2 purified Laccase from *Tp*FLU12; (**C**) Lane M: protein marker, Lane 1: In gel activity of purified Laccase from *Tl*FLU1, Lane 2: In gel activity of purified Laccase from *Tp*FLU12.

Table 1 shows the purification summary of Laccases from *Tl*FLU1 and *Tp*FLU12. Laccase from *Tl*FLU1 was purified to 25.3-fold showing specific activity of 4003 U/mg of protein and a total yield of 7.0% while Laccase from *Tp*FLU12 was purified to 5.6-fold showing specific activity of 790 U/mg of protein and a total yield of 5.5%. The purified Laccase from *Tl*FLU1 showed a single band with molecular mass of 44 kDa (Fig. 1a, Fig. S1), while *Tp*FLU-12 showed a molecular mass of 68.7 kDa (Fig. 1b, Fig. S2). The activity staining zymogram gel corresponding to the Laccase’s bands confirmed the nature of the enzymes (Fig. 1c, Fig. S3).

**Table 1:** Purification summary of Laccases purified from *Tl*FLU1 and *Tp*FLU12.

Strains	Purification step	Volume (mL)	Total activity (U)	Total protein (mg)	Specific activity (U/mg)	Yield (%)	Purification (fold)
*Tl*FLU1	Crude enzyme	50	21,198.8	134	158	100	1
	﻿(NH_4_)_2_SO_4_ precipitation (80%)	10	12,389.2	6.53	1897	58.4	12.0
	﻿Dialysis	20	8359.8	3.2	2612	39.4	16.5
	﻿Ion-Exchange Chromatography (DEAE-Cellulose)	10	4121.7	1.4	2944	19.4	18.6
	﻿Gel filtration on Sephadex G-100	5	1481	0.37	4003	7.0	25.3
	Crude enzyme	50	17,271	123	140	100	1
*Tp*FLU12	﻿(NH_4_)_2_SO_4_ precipitation (80%)	10	12,102	75.1	161	70.1	1.1
	﻿Dialysis	20	7287	21.97	332	42.2	2.4
	﻿Ion-Exchange Chromatography (DEAE – Cellulose)	7	1292	3.18	406	7.5	2.9
	﻿Gel filtration on Sephadex G-100	5	948	1.2	790	5.5	5.6

% Yield = relative to 21,198.8 considering 100%; Purification (fold) = relative to specific activity (U/mg) considering 1.

### UV–visible and FTIR spectra of purified Laccases

**Figure 2:**
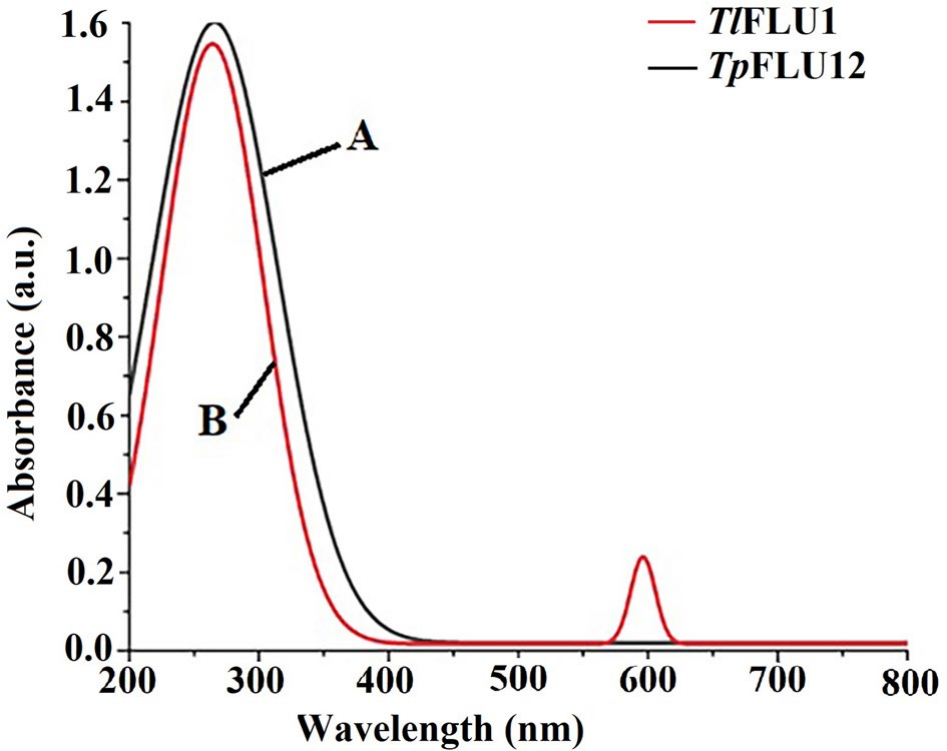
UV–vis spectrum of the purified Laccase from *Tl*FLU1 (curve A) and *Tp*FLU12 (curve B).

**Figure 3:**
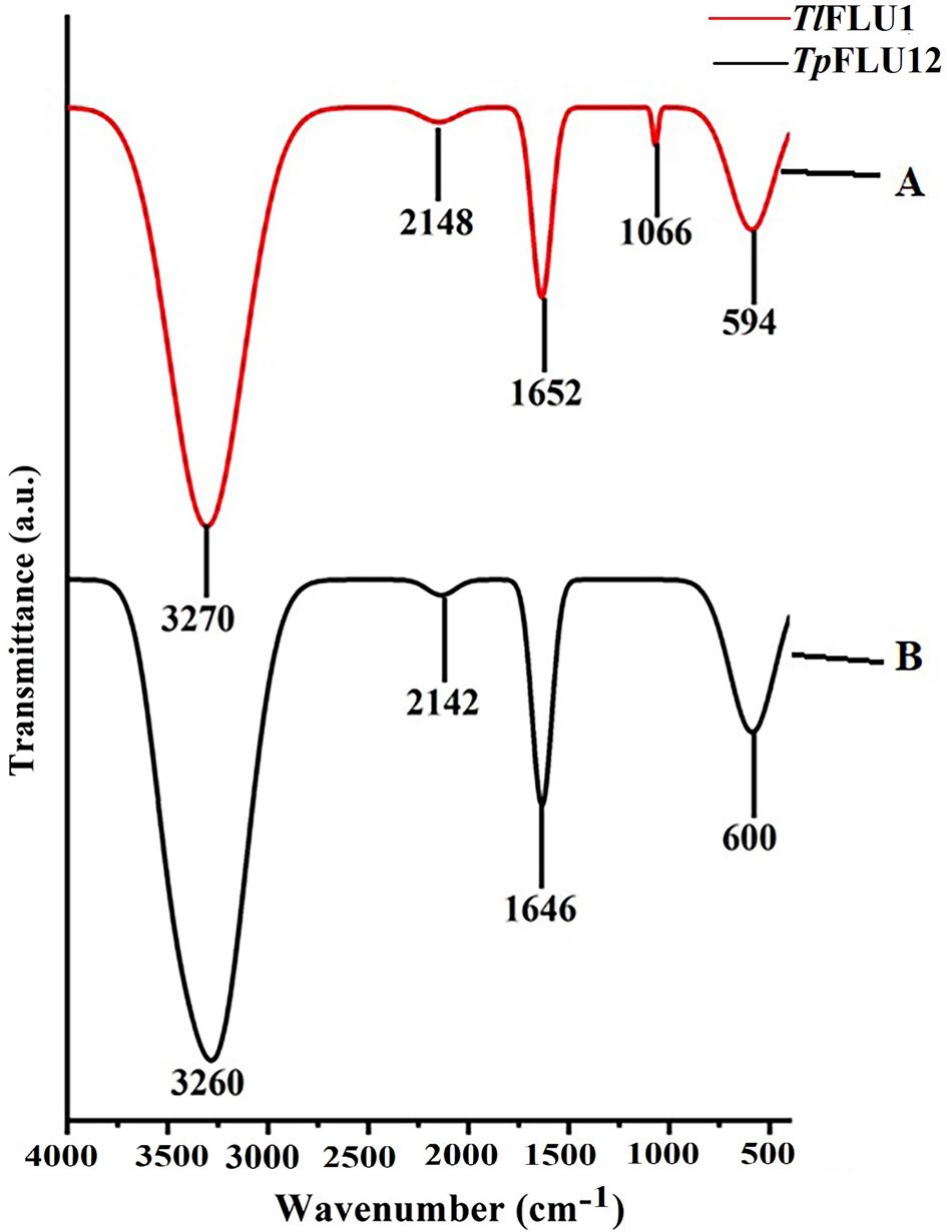
FTIR spectral of the purified Laccase from *Tl*FLU1 (curve A) and *Tp*FLU12 (curve B).

The purified Laccases from *Tl*FLU1 and *Tp*FLU12 showed the absorption peak at wavelengths 270 nm and 600, respectively, measured by UV–visible spectrophotometer (Fig. 2). The FTIR spectra of Laccase from *Tl*FLU1 showed the intense peaks at 3270 cm^−1^, 2148 cm^−1^, 1652 cm^−1^, 1066 cm^−1^ and 594 cm^−1^ attributing to –NH_2_ and –OH of amides A and B, OH/NH of Amide II, C=O stretching of α helix, C=C and C–N stretching of Amides II, respectively (Fig. 3, curve A, Table 2). The FTIR spectra of Laccase from *Tp*FLU12 showed the peaks at 3260 cm^−1^, 2148 cm^−1^, 1646 cm^−1^ and 600 cm^−1^ attributing to –NH_2_ and –OH of amides A, B, OH/NH of Amide II, ß-sheets of peptide and C=C stretching and C-N stretching of Amides II, respectively (Fig. 3, curve B, Table 2).

**Table 2:** Attribution of the infrared spectra absorbance bands of purified Laccase from *Tl*FLU1 and *Tp*FLU12.

Frequency (cm^−1^)	Attribution
> 3000	–NH_2_ and –OH of Amides A, B
2142–2148	OH/NH of Amide II
1600–1700	Amide I
1650–1658	C=O stretching of α helix
1620–1646	ß-sheets
594–1400	C=C stretching and C–N stretching of Amides II

### Optimum pH, optimum temperature and stabillity

**Figure 4:**
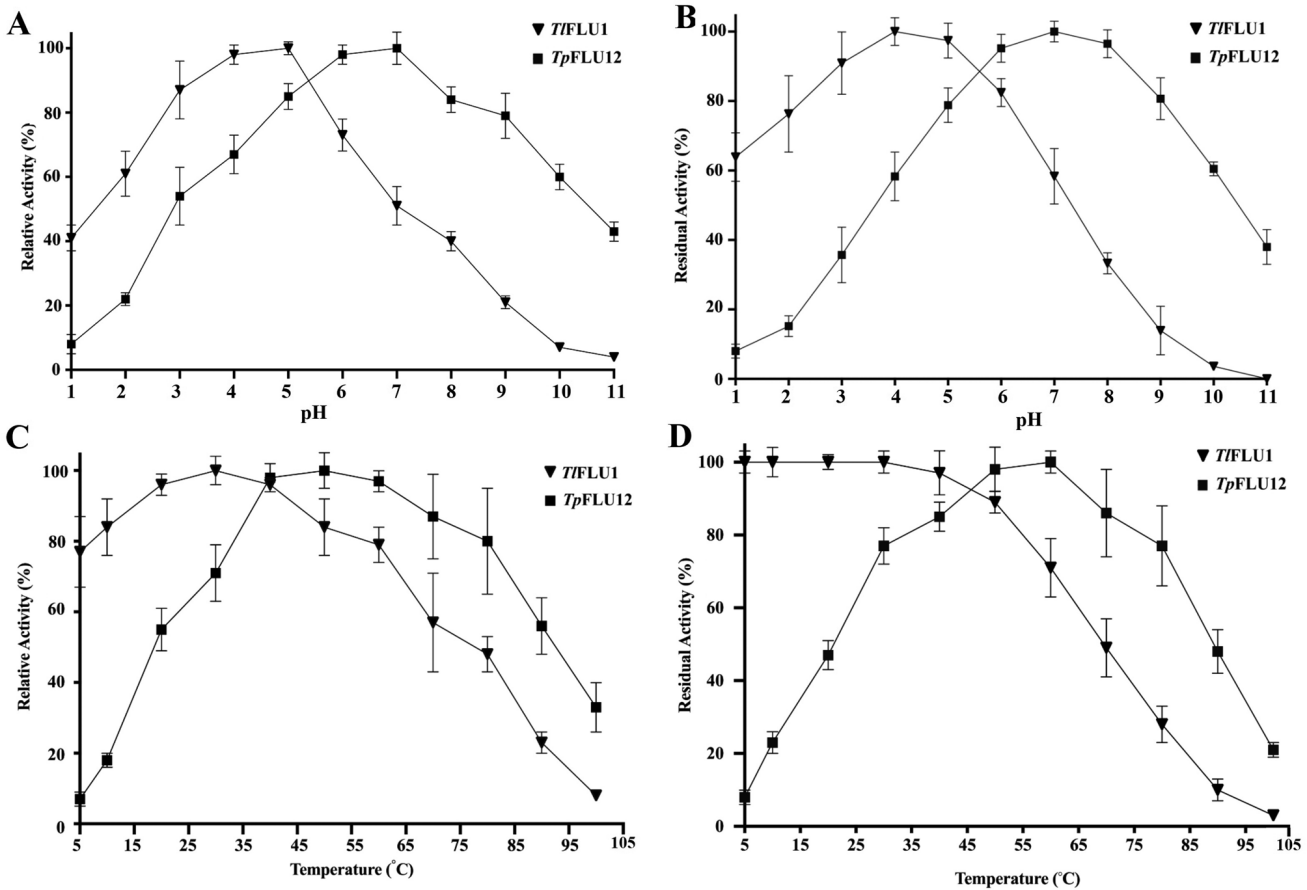
Optimum pH (**A**) and pH stability (**B**), optimum temperature (**C**) and temperature stability (**D**) of purified Laccase from *Tl*FLU1 and *Tp*FLU12.

Purified Laccases from *Tp*FLU1 and *Tp*FLU12 showed optimum activity at pH 5 and pH 7, respectively (Fig. 4a). *Tp*FLU1 Laccase showed 80–100% residual activity at pH 5–9 while *Tp*FLU12 showed 60–100% residual activity at pH 1–5 after 24 h of incubation period prior to the assay (Fig. 4b). Furthermore, Laccases from *Tp*FLU1 and *Tp*FLU12 showed optimum activity at temperatures 30 °C and 50 °C, respectively (Fig. 4c). After 24 h of incubation at 10–30 °C, Laccases from *Tp*FLU1 retained 100% activity and dropped to 90% at 50 °C, 70% at 60 °C, 50% at 70 °C and finally lost activity at 100 °C. *Tp*FLU12 Laccase residual activity was found to be very much stable between a temperature 50–60 °C. The enzyme showed 80% residual activity at temperatures 30 °C and 80 °C while retained 85% residual activity at 70 °C (Fig. 4d).

### Effect of metal ions, organic solvents and inhibitors on activity

Table 3 summarises the activity of Laccases in the presence of metal ions, organic solvents, and inhibitors. Laccase from *Tl*FLU1 showed 100%, 121%, 100%, 100%, 102%, 98.0% and 107% relative activity in the presence of Ca^2+^, Cu^2+^, Fe^2+^, Mg^2+^, Mn^2+^, Na^+^ and Zn^2+^, respectively, at 10 mM concentration, however, the activity was decreased with increased metal ion concentrations. Laccase from *Tp*FLU12 showed 104%, 100%, 101%, 119% and 100% relative activity in presence of Ca^2+^, Cd^2+^, Co^2+^, Cu^2+^and Na^+^ at concentration of 10 mM, while a relative activity of 100% was recorded in the presence of 50 mM Cu^2+^and Na^+^.

**Table 3:** Effect of metal ions on the activity of purified Laccase enzyme from *Tl*FLU1 and *Tp*FLU12.

Strains	Relative activity (%)			
	Ions	10 mM	50 mM	100 mM
*Tl*FLU1	Control	100.0 ± 0.64	100.0 ± 0.64	100.0 ± 0.64
	Al^3+^	37.0 ± 0.26	16.0 ± 0.15	10.0 ± 0.84
	As^5+^	80.0 ± 0.02	83.0 ± 0.17	62.0 ± 0.21
	Ca^2+^	100.0 ± 0.99	74.0 ± 0.77	22.7.0 ± 0.98
	Cd^2+^	70.0 ± 0.47	64.0 ± 0.27	42.0 ± 0.81
	Co^2+^	85.0 ± 0.64	87.0 ± 0.05	65.0 ± 0.16
	Cu^2+^	121.0 ± 0.93	84.0 ± 0.46	63.0 ± 0.88
	Fe^2+^	100.0 ± 0.15	33.0 ± 0.27	32.0 ± 0.97
	K^+^	33.0 ± 0.61	33.0 ± 0.32	30.0 ± 0.62
	Li^+^	46.0 ± 0.02	30.0 ± 0.18	25.0 ± 0.68
	Mg^2+^	100.0 ± 0.32	61.0 ± 0.05	48.0 ± 0.23
	Mn^2+^	102.0 ± 0.29	79.0 ± 0.54	18.0 ± 0.68
	Mo^+^	72.0 ± 0.81	70.0 ± 0.33	61.0 ± 0.38
	Na^+^	98.0 ± 0.36	96.0 ± 2.93	31.0 ± 4.95
	Ni^2+^	67.0 ± 0.37	81.0 ± 0.55	54.0 ± 0.19
	Zn^2+^	107.0 ± 0.21	89.0 ± 0.55	18.0 ± 0.79
*Tp*FLU12	Control	100.0 ± 0.66	100.0 ± 0.66	100.0 ± 0.66
	Al^3+^	75.0 ± 0.35	46.0 ± 0.39	27.0 ± 0.56
	As^5+^	83.0 ± 0.19	64.0 ± 0.41	79.0 ± 0.75
	Ca^2+^	104.0 ± 0.93	89.0 ± 0.22	70.0 ± 0.81
	Cd^2+^	100.0 ± 0.87	74.0 ± 2.17	41.0 ± 0.41
	Co^2+^	101.0 ± 0.65	75.0 ± 0.91	82.0 ± 0.17
	Cu^2+^	119.0 ± 076	100.0 ± 0.44	17.0 ± 0.58
	Fe^2+^	84.0 ± 0.81	57.0 ± 0.89	39.0 ± 0.65
	K^+^	73.0 ± 0.15	38.0 ± 0.53	16.0 ± 0.22
	Li^+^	74.0 ± 0.04	46.0 ± 0.74	19.0 ± 0.35
	Mg^2+^	82.0 ± 0.76	61.0 ± 0.27	49.0 ± 0.86
	Mn^2+^	81.0 ± 0.67	53.0 ± 0.41	34.0 ± 0.70
	Mo^+^	84.0 ± 0.49	76.0 ± 0.95	70.0 ± 0.13
	Na^+^	100.0 ± 0.81	100.0 ± 0.68	73.0 ± 0.82
	Ni^2+^	82.0 ± 0.25	66.0 ± 0.53	71.0 ± 0.18
	Zn^2+^	85.0 ± 0.75	73.0 ± 0.23	65.0 ± 0.23

Each value in the same column are the mean ± standard error.

The relative activity of Laccase enzyme from *Tl*FLU1 and *Tp*FLU12 in the presence of organic solvents is shown in Table 4. Enzyme from *Tl*FLU1 showed decreased relative activity with an increase in the organic solvents’ concentrations. 100% relative activity was recorded in the presence of acetone, acetonitrile, and ethyl-acetate while a relative activity of 127% was recorded in the presence of toluene at a concentration of 10%. The purified Laccase from *Tp*FLU12 was more active in organic solvents up to 90% of concentration. Maximum relative activity of 100% was recorded in the presence of acetonitrile and ethyl-acetate at a concentration of 10%. Relative activity of 100% was recorded in the presence of acetone and ethyl-acetate at a 50 and 90% concentration, respectively.

**Table 4:** Effect of organic solvents on the relative activity of purified Laccase enzyme from *Tl*FLU1 and *Tp*FLU12.

Strain	Organic solvent	Relative activity (%)		
		10%	50%	90%
*Tl*FLU1	Control	100.0 ± 0.71	100.0 ± 0.54	100.0 ± 0.49
	1,4-Dioxane	70.0 ± 0.34	69.0 ± 0.29	65.0 ± 0.24
	Acetone	100.0 ± 0.31	68.0 ± 0.53	43.6 ± 0.68
	Acetonitrile	100.0 ± 0.21	51.0 ± 0.24	19.0 ± 0.96
	Butanol	96.0 ± 0.85	88.0 ± 0.42	51.0 ± 0.25
	DMSO	66.0 ± 0.23	67.0 ± 0.83	41.7 ± 0.86
	Ethanol	73.0 ± 0.81	62.0 ± 0.14	51.5 ± 0.82
	Ethyl acetate	100.0 ± 0.42	73.0 ± 0.83	42.0 ± 0.67
	Isopropanol	89.0 ± 0.31	75.0 ± 0.27	61.3 ± 0.62
	Methanol	84.0 ± 0.63	77.0 ± 0.85	44.0 ± 0.77
	Toluene	127.0 ± 0.97	96.0 ± 0.5	53.4 ± 0.82
*Tp*FLU12	Control	100.0 ± 0.99	100.0 ± 0.65	100.0 ± 0.16
	1,4-Dioxane	93.0 ± 0.63	93.0 ± 0.13	44.1 ± 0.14
	Acetone	88.0 ± 0.20	100.0 ± 0.87	100.0 ± 0.45
	Acetonitrile	100.0 ± 0.03	97.0 ± 0.68	37.0 ± 0.68
	Butanol	70.0 ± 0.61	98.0 ± 0.79	82.0 ± 0.75
	DMSO	77.0 ± 0.93	99.0 ± 0.64	68.0 ± 0.92
	Ethanol	82.0 ± 0.61	89.0 ± 0.35	98.0 ± 0.77
	Ethyl acetate	100.0 ± 0.43	100.0 ± 0.82	100.0 ± 0.14
	Isopropanol	83.0 ± 0.51	97.0 ± 0.77	98.0 ± 0.17
	Methanol	76.0 ± 0.43	88.0 ± 0.52	97.0 ± 0.73
	Toluene	62.0 ± 0.71	83.0 ± 0.62	81.0 ± 0.63

Each value in the same column is the mean ± standard error.

**Figure 5:**
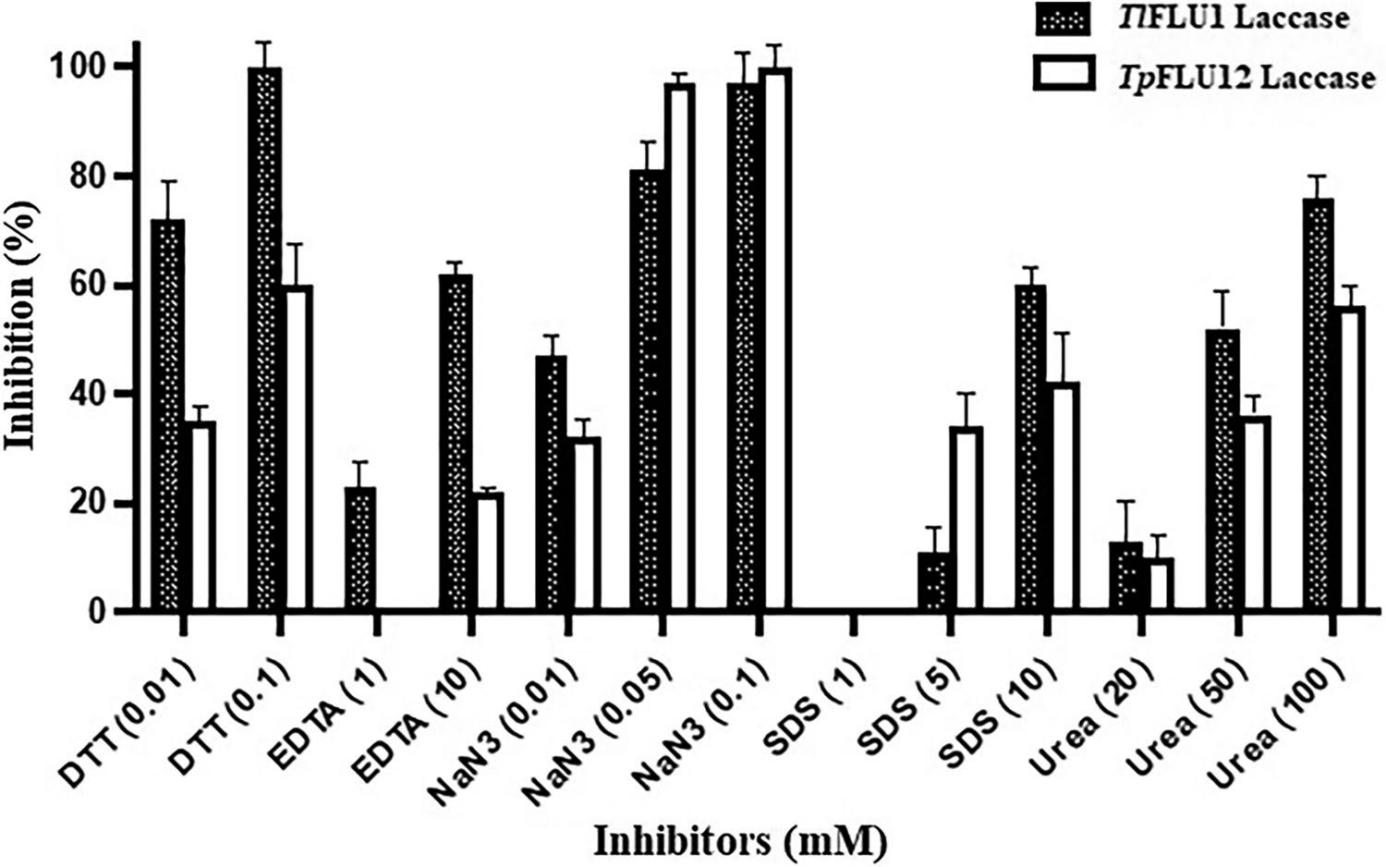
The percentage inhibition of inhibitors on Laccase activity from *Tl*FLU1 and *Tp*FLU12.

The enzymatic acvity of Laccase from both the isolates, *Tl*FLU1 and *Tp*FLU12 were significantly inhibited by the tested inhibitors (Fig. 5). NaN_3_ significantly inhibited Laccases from both strains showing 81% and 97% inhibition at a concentration of 0.05 and 0.1 mM for enzyme from *Tl*FLU1*.* In comparison, 97% and 100% inhibition were recorded at a concentration of 0.05 and 0.1 mM for enzyme from *Tp*FLU12*.*

### Kinetic parameters analysis

Table 5 shows the kinetics parameters values for Laccase from *Tl*FLU1 and *Tp*FLU12 when assayed with various substrates. Among all the phenolic compounds used as substrates, Laccase from *Tl*FLU1 and *Tp*FLU12 showed lowest *K*_m_ values 10.8 µM and 8.20 µM, for substrate ABTs, followed by guaiacol with *K*_m_ values of 12.7 µM and 11.6 µM while 13.03 µM and 14.91 µM for 2,6-DMP, respectively. Caffeic acid showed the lowest affinity as a substrate for both the enzymes from *Tl*FLU1 and *Tp*FLU12 with the *K*_m_ values of 43 µM and 38.60 µM.

**Table 5:** Kinetic characterization of purified Laccase from *Tl*FLU1 and *Tp*FLU12.

Strain	Substrate	Absorbance (nm)	ε (cm^−1^ mM^−1^)	*K*_m_ (µM)	*K*_cat_ (S^−1^)	*K*_cat_/*K*_m_ (µM^−1^S^−1^)
*Tl*FLU1	ABTS	420	36.00	10.80	88.25	8.17
	2,6-DMP	469	49.60	13.00	90.20	6.94
	Guaiacol	465	12.10	12.70	90.20	7.10
	Caffeic acid	312	11.20	43.00	116.20	2.70
*Tp*FLU12	ABTS	420	36.00	8.20	85.38	10.41
	2,6-DMP	469	12.10	11.60	89.17	7.69
	Guaiacol	465	49.60	14.90	92.20	6.19
	Caffeic acid	312	11.20	38.60	106.50	2.76

ε: Extinction coefficient.

### ES-MS analysis of tryptic digested proteins

ES-MS analysis of tryptic digested Laccase from *Tl*FLU1 and *Tp*FLU12 showed 12 and 18 protein clusters, respectively, when searched in UniProt Laccase database (Fig. S4a,b). The protein purified from *Tl*FLU1 showed 100% similarity with 386 amino acids long Laccase from *Trichoderma asperellum* strain OX with Uniprot accession number A0A6V8QJQ4 while protein purified from *Tp*FLU12 showed 100% similarity with 619 amino acids long Laccase from *Talaromyces marneffei* strain PM1 with Uniprot accession number A0A093UKS0 (Fig. S4c). Interestingly, no common proteins were detected between the Laccase from both strains at a 100% false discovery rate (FDR), and it quite raises concerns about relying soley on MS data sequence accuracy which might lead to unreliable models and inaccurate biophysical property predictions of the protein 3D structures. To address these limitations, a phylogenetic analysis using RSB PDB sequences of Laccases from both *Trichoderma* and *Talaromyces* families was conducted (Figs. S2 and S3). This approach provides a more robust understanding of the evolutionary relationships between the studied Laccases and their known counterparts, potentially offering a more reliable basis for modelling and prediction. Upon analysis, Laccase from *Tl*FLU1 was observed to share the same cluster with that of Laccase from *Trichoderma asperellum* with Uniprot accession number A0A6V8QJQ4 (Fig. S5), while Laccase from *Tp*FLU12 shared the same cluster with Laccase from *Talaromyces marneffei* PM1 with Uniprot accession number A0A093V1A3 (Fig. S6).

### Homology sequence alignment

The homology sequence alignment of Laccases from *Tl*FLU1 and *Tp*FLU12 were constructed with PDB template IGW0 (Laccase from *Melanocarpus albomyces*) (Fig. S7) and 4X4KA (Laccase from *Botrytis aclada*) (Fig. S8), respectively, using LOMETS threading programs of the I-TASSER server. Laccases from *Tl*FLU1 shared 13% sequence identity with the template IGW0 in the threading aligned region with the query sequence, 24% sequence identity of the whole template chains with the query sequence, 81% coverage of the threading alignment and 1.78 normalized Z-score of the threading alignments. Amino acids sequence WADNLINGYRLLNYWFNPDNPVNTLPNDPGVAVLNPRRWRHI (42 sequences) were observed as the consensus sequence between template IGW0. Laccase from *Tp*FLU12 shared 16% sequence identity with the template 4X4KA in the threading aligned region with the query sequence, 18% sequence identity of the whole template chains with the query sequence, 73% coverage of the threading alignment and 5.85 normalized Z-score of the threading alignments. Amino acids sequence DTGTHWHGQCPQPTCINLGDGFLINGFIDHLTVIANDVVARYTPPGSDNPLDDSKNNDNLNPSEITG (67 sequences) were observed as the consensus sequence between template 4X4KA.

### Template based structure prediction

**Figure 6:**
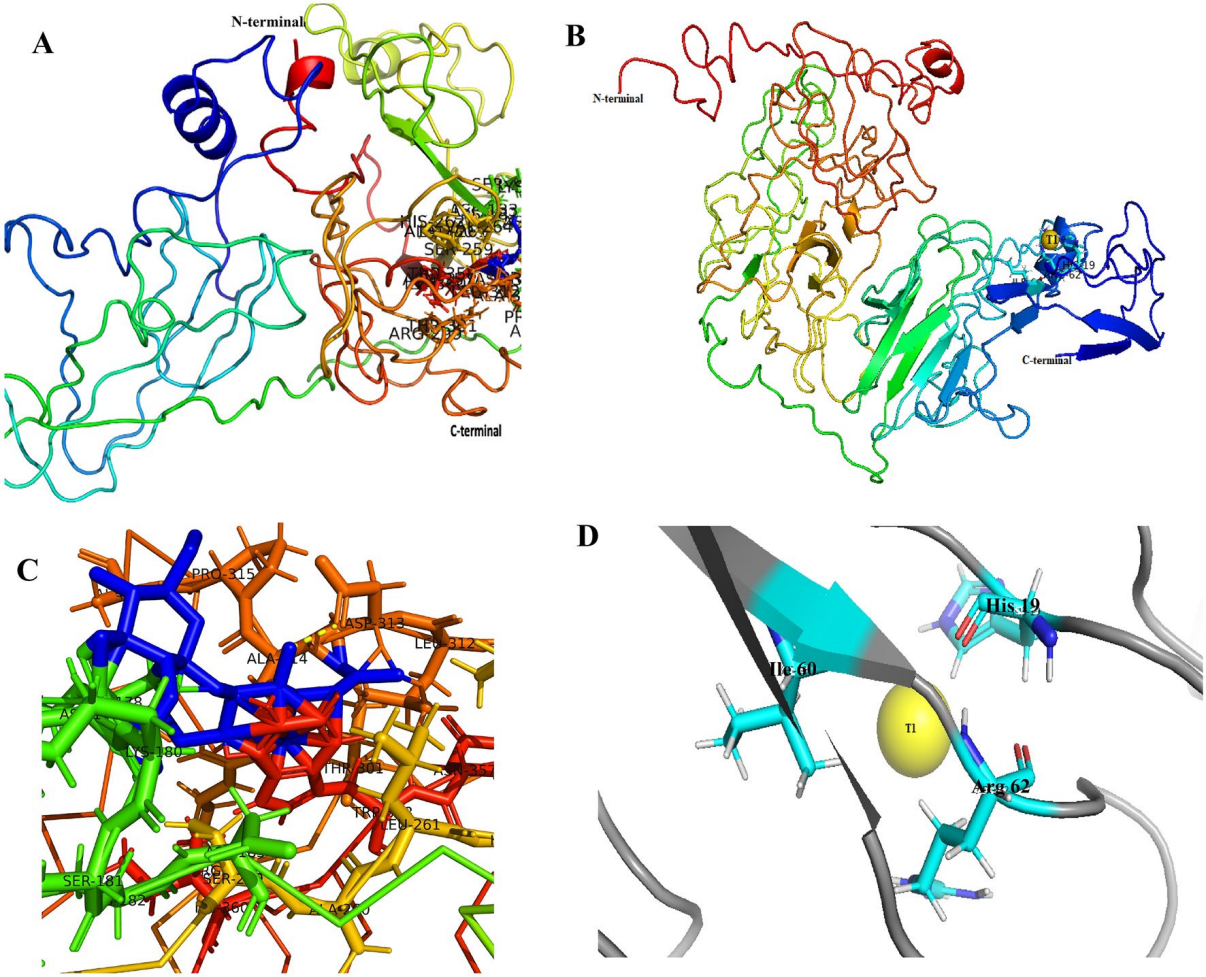
Structural models for Laccases from *Tl*FLU1 and *Tp*FLU12. Molecular surface structure with the binding sites (**A**) and (**B**); 3D cartoon (**C**) and (**D**); binding sites (**E**) and (**F**) of the Laccase from *Tl*FLU1 and *Tp*FLU12, respectively. The yellow ball colour (T1) is the Cu^2+^ of the protein.

Template-based structure modelling and ligand binding site residues of Laccases were predicted on iterative threading assembly refinement (I-TASSER) and shown in Fig. 6A–D and Table 6. The investigation revealed that the Laccase from *Tl*FLU1 shares 13% sequence identity with the IGW0 template within the threading-aligned region and exhibits 24% sequence identity with the complete template chains. Furthermore, the threading alignment covers 81% of the sequence and the normalized Z-score amounts to 1.78 (Fig. 6A). Likewise, the Laccase derived from *Tp*FLU12 shares 16% sequence identity with the 4X4KA template within the threading-aligned region and shows 18% sequence identity with the entire template chains. The threading alignment coverage encompasses 73% of the sequence, with a normalized Z-score of 5.85. Employing Jalview workbench alignment, 3-D models that conform to spatial restraints were generated, exhibiting high precision as evidenced by minimal restraint violations (Fig. 6B). Further, The I-TASSER server-generated predicted protein structures for *Tl*FLU1 and *Tp*FLU12, manifest a moderate degree of structural similarity based on the TM-score (0.48 ± 0.15 and 0.53 ± 0.15, respectively). However, these models exhibited moderate structural deviation, as indicated by the root mean square deviation (RMSD) values of 1.13 ± 0.5 Å and 1.15 ± 0.5 Å, respectively. Notably, the aligned sequences display a relatively low sequence identity (24% and 18%, respectively), implying a limited proportion of identical residues. Nevertheless, the alignment covered a substantial portion of the hit structure, as indicated by the coverage (Cov) values of 81% and 73%, respectively.

**Table 6:** The Laccase ligand binding sites residues and predicted functions.

Strain	Ligand binding Site residues	Consensus prediction of GO terms	GO terms code	GO terms functions	GO-score
*Tl*FLU1	Pro-177, Ala-178,	Molecular function	GO:0005507	Copper ion binding	0.65
	Asp-179, Lys-180,		GO:0016682	Oxidoreductase activity	0.36
	Lys-182, Asp-183,		GO:0008471	Laccase activity	0.34
	Ser-258, Ala-259,				
	Leu-260, His-261,	Biological process	GO:0055114	Oxidation—reduction	0.65
	Val-263, Arg-298,		GO:0009808	Lignin metabolism	0.4
	Thr-300, Leu-312,		GO:0046271	Phenylpropanoid catabolic	0.4
	Asp-313, Ala-314,		GO:0006725	Aromatic compound metabolism	0.4
	Pro-315, Als-316		GO:0030243	Cellulose metabolism	0.4
	Asn-355, Trp-356,				
	Ile-358	Cellular component	GO:0044421	Extracellular structure	0.4
*Tp*FLU12	His-19, Ile-60,	Molecular function	GO:0005507	Copper ion binding	0.44
	Arg-62		GO:0016724	Oxidoreductase activity	0.57
			GO:0052716	Oxygen oxidoreductase activity	0.46
		Biological process	GO:0055114	Oxidation—reduction	0.57
			GO:0022610	Biological adhesion	0.46
			GO:0044237	Cellular metabolism	0.44
			GO:0006875	Cellular metal ion homeostasis	0.44
			GO:0018955	Aromatic compound metabolism	0.44
		Cellular component	GO:0044421	Extracellular structure	0.4

To evaluate the quality of the models, verified 3D visualization using polymol was employed. The assessment revealed that the protein model derived from *Tl*FLU1 exhibited good 3D-1D scores for 43.95% of the total residues, while the model obtained for *Tp*FLU12 demonstrated good scores for 90% of the total residues. Structural analysis of the *Tl*FLU1 protein model unveiled the presence of four α-helices, with the absence of β-strands, β-α-β motifs, and β-hairpins. In contrast, the *Tp*FLU12 model displayed 104 α-helices, 60 β-sheets, one β-α-β motif, and two β-hairpins. Also, the Ramachandran plots (not shown) of the final minimized model suggested that the protein residues from *Tl*FLU1 was 9% most favourable, 12.11% additional allowed, 79% generously allowed and 0% disallowed, while that of *Tp*FLU12 had 72.7% most favourable regions, 17.3% additional allowed regions, 0% generously allowed regions and 0% disallowed regions. When compared with the template and results obtained from COACH and COFACTOR, 21 catalytic residues such as Pro-177, Ala-178, Aap-179, Lys-180, Lys-182, Asp-183, Ser-258, Ala-259, Leu-260, His-261, Val-263, Arg-298, Thr-300, Leu-312, Asp-313, Ala-314, Pro-315, Ala-316, Asn-355, Trp-356 and Ile-358 were obtained for Laccase from *Tl*FLU1 (Fig. 6C) while only Laccase from *Tp*FLU12 showed three combinations of catalytic residues namely His-19, Ile-60 and Arg-62 with Cu^2+^ (T1) residues at the binding site (Fig. 6D). The 21 ligands binding sites residues of Laccase from *Tl*FLU1 were shown to be associated with molecular functions related to copper ion binding, oxidoreductase activity and Laccase activity; biological process related to oxidation–reduction, lignin metabolism, phenylpropanoid catabolic, aromatic compound metabolism and cellulose metabolism; and cellular component related to extracellular structure; according to the gene ontology (GO). There were 3 binding residue sites (His-19, Ile-60 and Arg-62) of Laccase from *Tp*FLU12 were predicted with molecular functions related to copper ion binding, oxidoreductase activity and oxygen oxidoreductase activity; biological process related to oxidation–reduction, biological adhesion, cellular metabolism, cellular metal ion homeostasis, aromatic compound metabolism; and cellular component related to extracellular structure (Table 6).

### Biophysical properties

The amino acid sequences (386 and 619 amino acids for Laccases from *Tl*FLU1 and *Tp*FLU12, respectively) were submitted to ProtParam to predict their biophysical properties (Table 7). Molecular mass of 43.8 and 68.6 kDa, theoretical pI value of 9.33 and 5.41 were predicted for Laccases from *Tl*FLU1 and *Tp*FLU12, respectively. The extinction coefficient values of 68,995 M^−1^cm^−1^ and 85,050 M^−1^cm^−1^ assuming all pairs of Cys residues form cystines while 68,870 M^−1^cm^−1^ and 84,800 M^−1^cm^−1^ when assuming all Cys residues are reduced were exhibited by Laccase from *Tl*FLU1 and *Tp*FLU12, respectively. However, Laccase from *Tl*FLU1 was predicted to have an estimated half-life of 4.4 h in mammalian reticulocytes (in vitro), > 20 h in yeast (in vivo) and > 10 h in *E. coli* (in vivo) while that of *Tp*FLU12 Laccase was estimated to have a half-life of 1.1 h in mammalian reticulocytes (in vitro), > 3 min in yeast (in vivo) and > 10 h in *E. coli* (in vivo), confirming their stability in eukaryotic and prokaryotic cells. The predicted instability, aliphatic index, and grand average of hydropathicity (GRAVY) values of 39.54, 33.43; 76.58, 77.50 and -0.508, -0.578 for Laccases from *Tl*FLU1 and *Tp*FLU12, respectively, classify them as stable, thermophilic, and non-polar.

**Table 7:** The Biophysical and chemical properties of Laccase from *Tl*FLU1 and *Tp*FLU12.

Property	*Tl*FLU1	*Tp*FLU12
Number of amino acids	386	619
Molecular weight (kDa)	43.8	68.6
Theoretical pI	9.33	5.41
Extinction coefficients (M^−1^ cm^−1^)		
Cys residues from cystines	68,995	85,050
Cys residues are reduced	68,870	84,800
Estimated half-life		
Mammalian reticulocytes (In-vitro)	4.4 h	1.1 h
Yeast (In- vivo)	> 20 h	3 min
* E. coli* (In-vivo)	> 10 h	> 10 h
Instability index	39.54 (stable)	33.43 (stable)
Aliphatic index	76.58 (Thermophilic)	77.50 (Thermophilic)
Grand average of hydropathicity (GRAVY)	− 0.508 (hydrophilic)	− 0.578 (hydrophilic)

### Degradation of PAHs by purified Laccase

**Figure 7:**
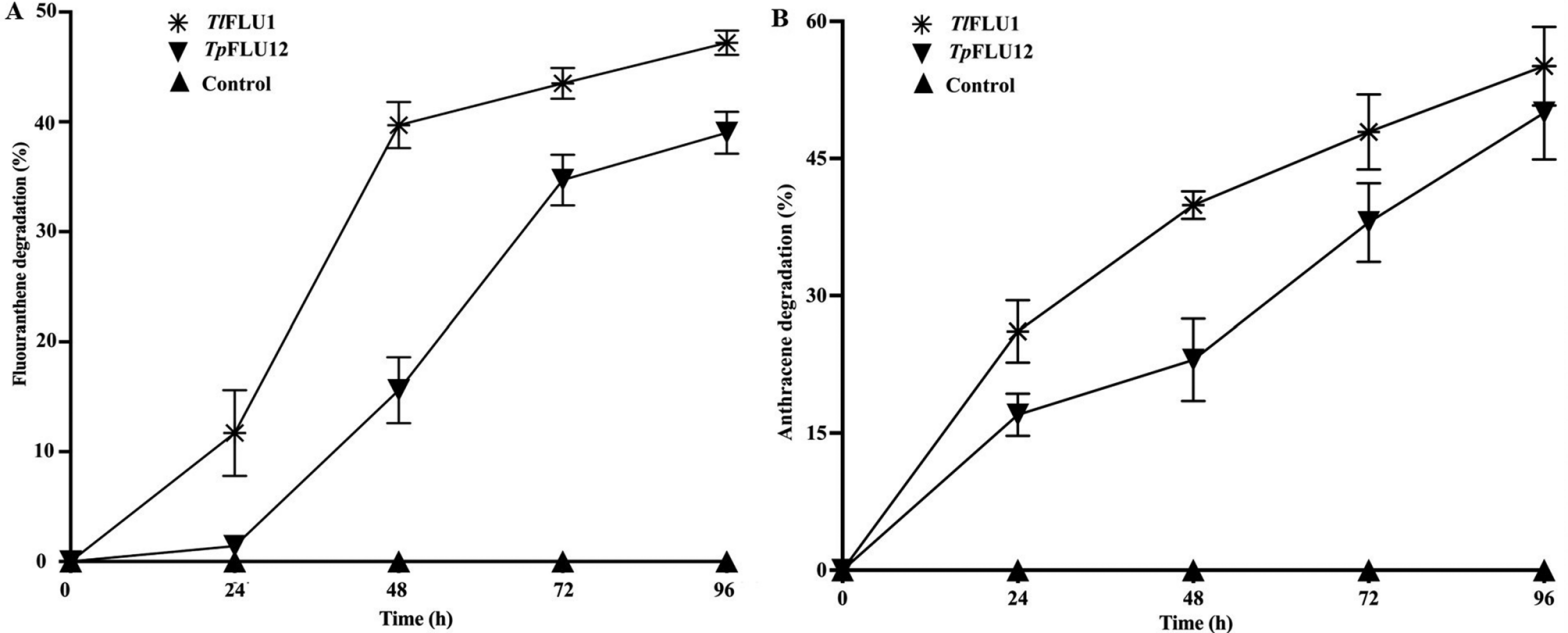
Biotransformation of PAHs fluoranthene (**A**) and anthracene (**B**) by purified Laccase from *Tl*FLU1 and *Tp*FLU12. The % transformation of fluoranthene and anthracene is 100% residual concentration. Control experiment curves are overlapping the x-axis.

A general reduction in the residual PAHs (Fluoranthene and anthracene) concentrations were observed after 96 h incubation period (Fig. 7A,B). For fluoranthene, a residual concentration of 61.0% of 200 mg/L initial concentration was recorded in *Tl*FLU1 Laccase incubated tube while that of the *Tp*FLU12 Laccase incubated tube accounted for 52.8% residual fluoranthene concentration in comparison with the control tube (no Laccase addition) where no reduction was recorded in its initial concentration (100%). Therefore, 39.0% and 47.2% transformation of fluoranthene was recorded by Laccases from *Tl*FLU1 and *Tl*FLU12, respectively (Fig. 7A). Also, during anthracene degradation, a residual concentration of 44.9% was recorded at 96 h incubation period in the *Tl*FLU1 Laccase incubated tube while that of the *Tp*FLU12 Laccase incubated tube showed a residual concentration of 50.0% at the same incubation period in comparison with the control tube (no Laccase addition) where no reduction was recorded in its initial concentration (100%). There was 55.1% and 50.0% transformation of fluoranthene was recorded by Laccases from *Tl*FLU1 and *Tl*FLU12, respectively (Fig. 7B).

## Discussion

The capability of *Tl*FLU1 and *Tp*FLU12, investigated in this study, to produce active Laccases with a robust cold-adaptation and thermostability using anthracene as the sole carbon source corroborates previous study^33^, where the use of anthracene as a sole carbon source for growth and production of Laccase with high pH stability and temperature stability in an ascomycete fungus, *Fusarium solani* MAS2.

In this study, the obtained low purification fold with high protein-specific activities of purified Laccases from both strains following adoption of chromatographic techniques like ion-exchange (DEAE-cellulose) and gel filtration (Sephadex G-100) in chronological order is an indication that Laccases were the most abundant among the extracellular enzymes released into the media^43^. Similarly, the purification fold falls within the range of a previously reported purified Laccase from *Ganoderma lucidum* with a purification fold of 25.4^44^. The absence of a peak near 600 nm in the UV–vis spectrum at 270 nm observed in Laccase from *Tl*FLU1 suggests the presence of a type 2 copper centre with unique Laccase peptide interactions, which is characteristic of white Laccase enzymes^45^. Also, the absence of type 1 spectrum (blue Laccase) could be due to incomplete oxidation of copper ions which then results in the formation of one copper atom at the catalytic site in combination with metal ions like zinc, iron or manganese, which give it four active reaction sites^46^. Similarly, the presence of white/yellow Laccase in ascomycete fungi might may be due to the quenching of the type 1 Laccase (blue Laccase) through an exogenous bond of aromatic group harnessed from the carbon source used during production, which then acts as a redox mediator in its high redox potential^25^. In addition, the UV–vis spectrum absorption at 600 nm by Laccase from *Tp*FLU12 is a characteristic of blue Laccase due to the type 1 spectrum peak^24^. Fourier-transform infrared spectroscopy (FTIR) analysis is an effective tool used in analysing proteins and polypeptides structures. In *Tp*FLU-12 Laccase, the observed vibrational bands at wavenumber 1646 cm^−1^ attributing to β-sheets, absorption at 600 cm^−1^ (amides II) and amide A, B (bands > 3000 cm^−1^) is an indication of blue Laccase^24^ while the peaks at 594–1066 cm^−1^, 1652 cm^−1^ and > 3000 cm^−1^ in purified Laccase from *Tl*FLU1 could be linked to α helix, amides II and amide A, B of white Laccase structures^45^. It is important to note that the peaks at 2142 cm^−1^ and 2148 cm^−1^ observed in both purified Laccases represent the presence of O–H stretching due to water interaction with the protein and its polypeptide structures.

Under denaturing and native conditions, the single band of both Laccases with a molecular mass of 44 and 68.7 kDa from *Tl*FLU1 and *Tp*FLU12, respectively, is consistent with other documented fungal Laccases (40–66 kDa) (Kumar et al. 2017). A Laccase with molecular mass of 45 kDa have been previously reported to be produced by *Ceriporiopsis subvermispora* (Jung et al. 2002). Another Laccases showing a molecular mass of ~ 67kD^47^ and with a molecular mass of 70 kDa from ascomycete fungus, *Thielavia sp.* HJ22^48^ are also reported previously, very close to Laccase purified from *Tp*FLU12 (68.7 kDa) in this study.

In this study, the catalytic rate of Laccases and their stability were significantly influenced by pH and temperature which is of industrial advantage compared to previous reports. The optimum pH activity (5 and 7) and stability at a wide range of pH for both Laccases corroborate with the previous study^49^ where optimum pH activity of white Laccase was reported at pH 4 and stable from pH 2–7. Similarly, relative activities of two Laccase isoforms (Lacc1 and Lacc2) from *﻿Agaricus bisporus* CU13 were optimum at pH of 5 and 7, respectively, while the stability was reported at pH 7 and pH 9^50^. Here, optimum temperature of 30 °C and 50 °C were recorded for Laccase from *Tl*FLU1 and *Tp*FLU12, respectively, is corroborated with previous report^51^ where a temperature of 30 °C best-enhanced Laccase activity of a Laccase from white-rot fungus *Trametes hirsute* and maintained its 80% activity at a temperature between 20 to 40 °C. Likewise, an optimum temperature of 50 °C has been implicated in enhancing the maximum activity for purified Laccase from *Lentinus tigrinus*^52^. The temperature stability of purified Laccase from *Tl*FLU1 (white Laccase), which is from 5 to 50 °C with over 90% residual activity after 24 h incubation period, is inconsonant with the previous report^53^, where thermo-stability reported between 50 and 70 °C for Laccase from *Trichoderma harzianum* strain HZN10﻿. Notably, the observed difference in Laccase activity between *Tl*FLU1 and *Tp*FLU12 at 5 °C could be attributed to the inherent genetic and physiological differences between the two strains^54–56^. *Tl*FLU1 might have a more robust cold-shock response mechanism that allows it to maintain higher Laccase activity at lower temperatures compared to *Tp*FLU12. This aligns with a previous report, where Laccase from *Thielavia terrastris* Co3Bag1 retains 86% of its activity after 12 d storage at 4 ˚C due to cold-shock response^57^. Similarly, it has been reported that Laccases from fungi can retain activity at low temperature (4–7 °C)^58^. Conversely, the substantial loss of activity in *Tp*FLU12 below 20 °C after 24 h of incubation could be since Laccase like many enzymes, has an optimal temperature range for activity^59^. At higher temperatures, fungi Laccases have been reported to possess characteristics of retaining more activity above 20 °C after 24 h^57^. Also, Psychrophilic Laccases, such as those produced by *Kabatiella bupleuri* G3 IBMiP which have been documented to have exhibit an optimal temperature range of 30–40 °C, with a significant decrease in activity below this ranges but still retains some activity even at 10 °C. This behaviour further attests to the substantial loss of activity in *Tp*FLU12 below 20 °C after 24 h of incubation period.

The effect of different metal ion concentrations on Laccase stability is highly imperative in assessing the enzyme’s biotechnological applications. The significant low activity of Laccase from *Tl*FLU1 in the presence of metal ions (Al^3+^ and K^+^) at a low concentration (10 mM) implies that these metal ions are activity inhibition stimulator which interferes with the electron transport system of Laccase activity. Similarly, a previous report linked Laccase inhibition with interaction of Fe^2+^ with the electron transport system of Laccase^60^. Laccase from *Ganoderma lucidum* showed complete activity inhibition in the presence of Fe^2+^ during the effect of metal ions on reactive dye decolourization^61^*.* 1 mM K^+^ reported to show 54% inhibition activity of Laccase from *Lentinula edodes*^62^. A detailed study on the inhibitory effect of metal ion concentration of 12.5 mM showed that Al^3+^ inhibit 55.9% and Mn^2+^ inhibit 63.5% Laccase activity on Laccase activity^63^. It is worth noting that the significant decrease in activity of Laccase from *Tl*FLU1 at concentrations above 10 mM suggests that Laccase activity is a function of the metal ions concentration^63^. Conversely, the increase in activity of Laccase from *Tp*FLU12 in the presence of high Ca^2+^, Cd^2+^, Co^2+^, Cu^2+^ and Na^2+^ concentrations suggest them as a potent Laccase activity activator due to the oxidation state (divalent cations) of the metal ions. It is opined that divalent cations of metal ions enhance Laccase activity and stability which aided in its binding to the carboxylic group of aspartic and glutamic acid residues for better complete substrate consumption and catalytic effect activation^64^. Also, the stability of Laccase activity to a high concentration of Cu^2+^ (100 mM) might be due to the type-2 copper-binding sites filled by the copper ions^62^.

The phenomenon of Laccase activity stability at a varying concentration of hydrophobic and hydrophilic organic solvents in this study could be ascribed to the stripping-off of the crucial bound-water monolayer from the enzyme molecule^64^. Also, the variability in the stability of purified Laccase from *Tp*FLU12 observed at high organic solvents concentrations could be linked to each solvent's physical and chemical properties, the mixture pH, log P of the solvent and thermodynamic activity of water in mixed systems^23,65^. Unlike Laccase from *Tp*FLU12, Laccase from *Tl*FLU1 could not tolerate low water content systems containing organic solvents, which explains why high organic solvent concentrations inhibited its stability. A similar observation has been reported previously^66^, where Laccase stability was inhibited at high organic solvent concentrations after an hour incubation period which was credited to a low water content of the solvent.

The extent of the inhibitory effect of ﻿metalloenzymes and detergents varied greatly with their concentrations. The high inhibitory effect of NaN_3_ in both strains is due to internal electron transfer obstruction at the types 2 and 3 Laccase copper atom binding sites^65^. The high inhibition rate of the ﻿chelating agent (EDTA), denaturing agents DDT and SDS at varying concentrations could be ﻿justified by their interactions between the substrate and the Laccase active site leading to the unfolding of the compact protein structure during the catalytic enzyme activity^48^. Likewise, the high inhibition by NaN_3_ and EDTA is due to the chelating effect on the Cu atoms, which leads to inactivation of the Laccase^12^, while high inhibition by ﻿DTT suggests the strong inhibitory effect on the di-sulphide bonds present at the Laccase active domain^33^.

The obtained *K*_m_ and *K*_cat_ values established the superior affinity of the purified Laccases towards substrate ABTS as compared to other phenolic substrates (2,6 DMP, caffeic acid and guaiacol). This observation agrees with the previous reports^48^, where strong affinity towards ABTS as substrate was documented during the characterization of Laccase from an ascomycetes fungi, *Thielavia sp.* In comparison with that of Laccase from *Tp*FLU12 (blue Laccase), the observed high *K*_m_ and *v*_max_ values for purified Laccase of *Tl*FLU1 (white Laccase) in the presence ABTS, 2,6 DMP, caffeic acid and guaiacol confirm the characteristics of a yellow Laccase of fungi origin^24,44^.

The ES-MS generated unique Laccase peptides from Laccases from *Tl*FLU1 and *Tp*FLU12 and showed different numbers of protein clusters suggesting that these ascomycetes fungi produce two types of Laccases. The most likely explanation for this observation is that the number of Cys residues in the putative copper-binding site in the peptide sequence leads to the formation of two disulphide bridges which are known to be titratable in non-reducing conditions, a sole characteristic type-I copper (blue Laccase)^67^. Also, changes in the valence state of the Laccase Cu^2+^ residues and Fe^2+^ residues with a low spin electronic configuration account for the lack of blue colouration in white Laccase from *Tl*FLU1 could be attributed to the differences in the numbers of protein clusters and unique Laccase peptides^49^. The variation in ascomycetes amino acids sequence number and structural differences in sensu stricto Laccases is linked to variation in the number of their encoding genes^68^.

Sequence homology analysis according to the LOMETS threading programs of the I-TASSER server revealed that variation in consensus sequence of Laccases from *Tl*FLU1 and *Tp*FLU12 which ranges between 42 and 67 suggests these sites as a major contributing factor to the protein stability due to non-covalent interactions^69^. Similarly, a previous report, demonstrated how 20 consensus mutation sequence cloned into *Saccharomyces cerevisiae* was linked to stabilizing Laccase with high-redox without compromising its catalytic activity^70^.

Prediction of binding site residues such as Pro-177, Ala-178, Aap-179, Lys-180, Lys-182, Asp-183, Ser-258, Ala-259, Leu-260, His-261, Val-263, Arg-298, Thr-300, Leu-312, Asp-313, Ala-314, Pro-315, Ala-316, Asn-355, Trp-356 and Ile-358, His-19, Ile-60 and Arg-62 in Laccases from both strains further strengthen the result of this study that the purified enzyme is Laccase with high catalytic activity since their high conservation across the amino acid sequences have been linked in the stabilization of the protein secondary structure leading to correct formation of the copper-binding site^71^. Similarly, it is opined that positively charged amino acids like Arg), His and Lys are responsible for the favourable conformation changes in Laccase leading to an increase in its catalytic activity^69^. It is previously demonstrated how binding residues such as Ala191, Pro192, Glu235, Leu363, Phe371, Trp373, Phe427, Leu429, Trp507 and His508 are crucial in the oxidation of phenolics during the structure–function studies of *Melanocarpus albomyces* Laccase^72^. Additionally, the Laccase gene ontology predicted molecular functions such as copper ion binding, oxidoreductase activity and Laccase activity, biological processes such as oxidation–reduction, lignin metabolism, phenylpropanoid catabolic, biological adhesion, cellular metabolism, cellular metal ion homeostasis, aromatic compound metabolism and cellulose metabolism and cellular component such as extracellular structure is not surprising because Laccases are known as oxidoreductases^68^, with Cu atoms in the active site, capable of lignin metabolism^73,74^, phenylpropanoid^75^, biological adhesion^74^, aromatic compound metabolism like fluorene^76^, cellular metal ion homeostasis^77^ and cellulose metabolism^78^.

The TM-score (0.48 ± 0.15 and 0.53 ± 0.15), and RMSD values (1.13 ± 0.5 Å and 1.15 ± 0.5 Å) observed in the model structure prediction for Laccases from *Tl*FLU1 and *Tp*FLU12, respectively, indicted a moderate degree of structural similarity with the PDB hit template used for model prediction since TM score close to 1 is classified as a perfect match with template structure, while a lower RMSD indicates a better structural alignment or match with template structure^36,37^. Despite covering a substantial portion of the hit structure, as indicated by the coverage (Cov) values of 81% and 73% for Laccases from *Tl*FLU1 and *Tp*FLU12, respectively, the aligned sequences displayed a relatively low sequence identity (24% and 18%). The most probable explanation for this observation can be linked to evolutionary divergences between the taxa of the template used and that of Laccases from *Tl*FLU1 and *Tp*FLU12^79^. Similarly, a previous study opined further that as species diverge evolutionarily, the sequence identity between their protein-coding regions may decrease due to changes in selective pressures and the fixation of different alleles^80^. Further, verify3D is a commonly used method for evaluating the quality of protein models. It compares the 3D environment of each residue in the model with high-resolution structures, assigning a 3D-1D score based on compatibility^81^. The predicted protein structure model obtained a high 3D-1D score in this study, indicating its excellent quality and similarity in overall shape and active site with the template structure^82^. The underlying assumption of verify3D is that a good 3D-1D score implies a similar structural arrangement to well-established protein structures^83^. Therefore, if the model's residues exhibit comparable structural characteristics and environments to those in known high-quality structures, it suggests that the model itself is likely of good quality.

Bioinformatics analysis by ProtParam further lends credence to this study finding that the two strains possess two different Laccase types. The predicted molecular weight of 44 and 68.6 kDa and theoretical pI values 5.22 and 6.32 agree with previous studies, where Laccase amino acids sequence of 566–600 aa, a molecular weight of 61.83–66.84 KDa and pl of pH 4–6 have been documented in *Trichoderma* Laccases^68^. While an amino acids sequence of 594 and with a molecular weight of 66.6 kDa close to this study has been reported in *Talaromyces* *marneffei* (strain ATCC 18,224/CBS 334.59/QM 7333). The predicted low instability indices (39.54 for *Tl*FLU1 and 33.43 for *Tp*FLU12) further gave an insight that the enzyme could be stable under harsh conditions. This prediction agrees with the previous report^84^ where low instability indices of 31.92 was linked to the Laccase from *Kurthia huakuii* stability under harsh conditions. Similarly, a report^85^ documented how low Laccase instability indices of 34.70 close to this study's prediction gave 40% of the Laccase relative activity at 70 °C after 60 min incubation period. In addition, the predicted aliphatic index values of the Laccase proteins ranged from 76.58 to 77.50 with a GRAVY index values mostly negative suggesting this protein to be thermophilic and hydrophilic nature. This prediction is in corroborated with previous reports^86^ where aliphatic index values of the Laccase proteins ranged from 74.44 to 90.4 with negative values of GRAVY index were attributed to the thermophilic and hydrophilic nature of Laccase during the comparative modelling and molecular docking analysis of white, brown and soft rot fungal Laccases using lignin model compounds for understanding the structural and functional properties of Laccases.

The findings of this study suggest that Laccase enzymes from *Tl*FLU1 and *Tp*FLU12 have the potential to degrade fluoranthene and anthracene, two commonly encountered PAHs of environmental concern. This observation is consistent with previous research highlighting the ability of fungi Laccase (50 U) purified from *Leucoagaricus gongylophorus* in degrading 10 ppm of anthracene (72 ± 1%), fluorene (40 ± 3%) and phenanthrene (25 ± 3%), respectively at pH 6 and 30 °C under 24 h incubation period^28^. Similarly, a previous study demonstrated the degradation of chrysene (75.8%) and Benzo[a]pyrene (35.6%), using a Laccase from *Trametes versicolor*^29^.

## Conclusion

The study demonstrated the utilization of anthracene as carbon and energy sources by *Tl*FLU1 and *Tp*FLU12, resulted in the production of Laccase enzymes with broad catalytic potentials. The enzymes with molecular mass of 44 kDa and 68.7 kDa respectively, exhibit different optimum pH and temperature ranges with an implication potential application across various conditions. Despite the differences, both enzymes showed an enhanced activity under diverse metal ions and organic solvents. Structural predictions suggest that the differing in the copper atoms in the active sites contribute to their varied optimal pH. Additionally, the functional analysis indicates their involvement in key biological processes such as oxidation–reduction, lignin metabolism and aromatic compounds metabolism. This study provides valuable insights into the potential of Laccase-based systems for PAH removal and highlighting the potential of these enzymes as eco-friendly alternative for xenobiotic degradation.

### Supplementary Information

Supplementary Information.

## Data Availability

Data is provided within the manuscript or supplementary information files.
