# Supplementary material for: Purification, characterization and three-dimensional structure prediction of multicopper oxidase Laccases from Trichoderma lixii FLU1 and Talaromyces pinophilus FLU12
Source: Sci Rep. 2024 Jun 11;14:13371. doi: 10.1038/s41598-024-63959-z (PMC11167041; doi:10.1038/s41598-024-63959-z)
Supplement: Supplementary file 1 — Supplementary Information. [file 41598_2024_63959_MOESM1_ESM.pdf]

SUPPLEMENTARY MATERIALS

Journal: Scientific Reports

Purification, Characterization and Three-Dimensional Structure Prediction of Multicopper Oxidase Laccases from *Trichoderma lixii* FLU1 and *Talaromyces pinophilus* FLU12

Samson O. Egbewale, Ajit Kumar, Mduduzi P. Mokena, Ademola O. Olaniran*

Discipline of Microbiology, University of KwaZulu- Natal (Westville Campus), Durban, Republic of South Africa.

* Corresponding author. Tel. +27 31 260 7400

Fax: +27 31 260 7809

Email address: olanirana@ukzn.ac.za

Figure S1: Raw gel for figure 1a. Area shown in the box was cropped and presented as Figure 1a.

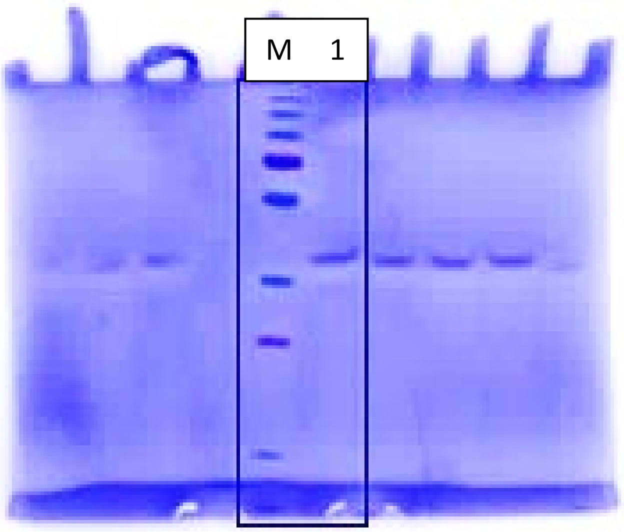

Figure S2: Raw gel for figure 1b. Area shown in the box was cropped and presented as Figure 1b.

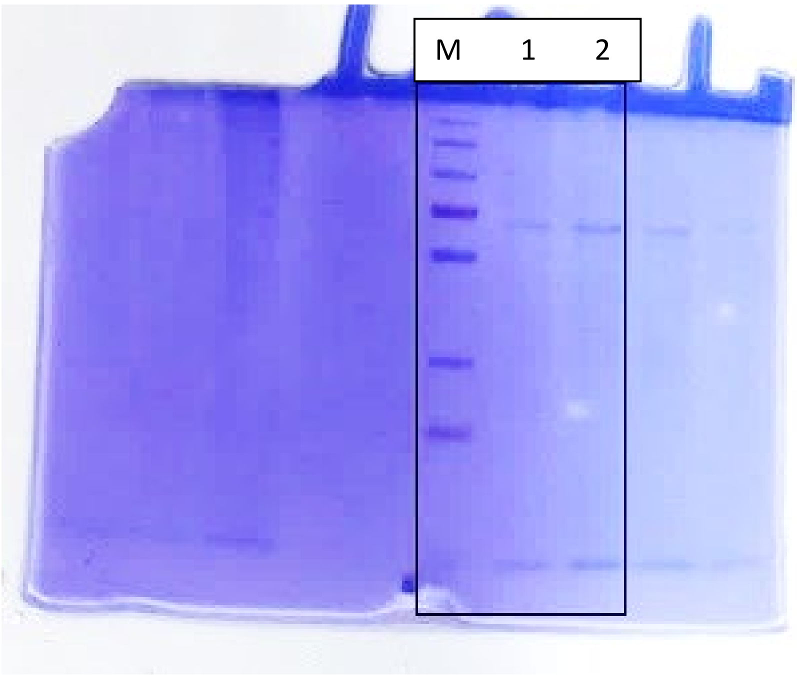

Figure S3: Raw gel for figure 1c. Area shown in the box was cropped and presented as Figure 1c.

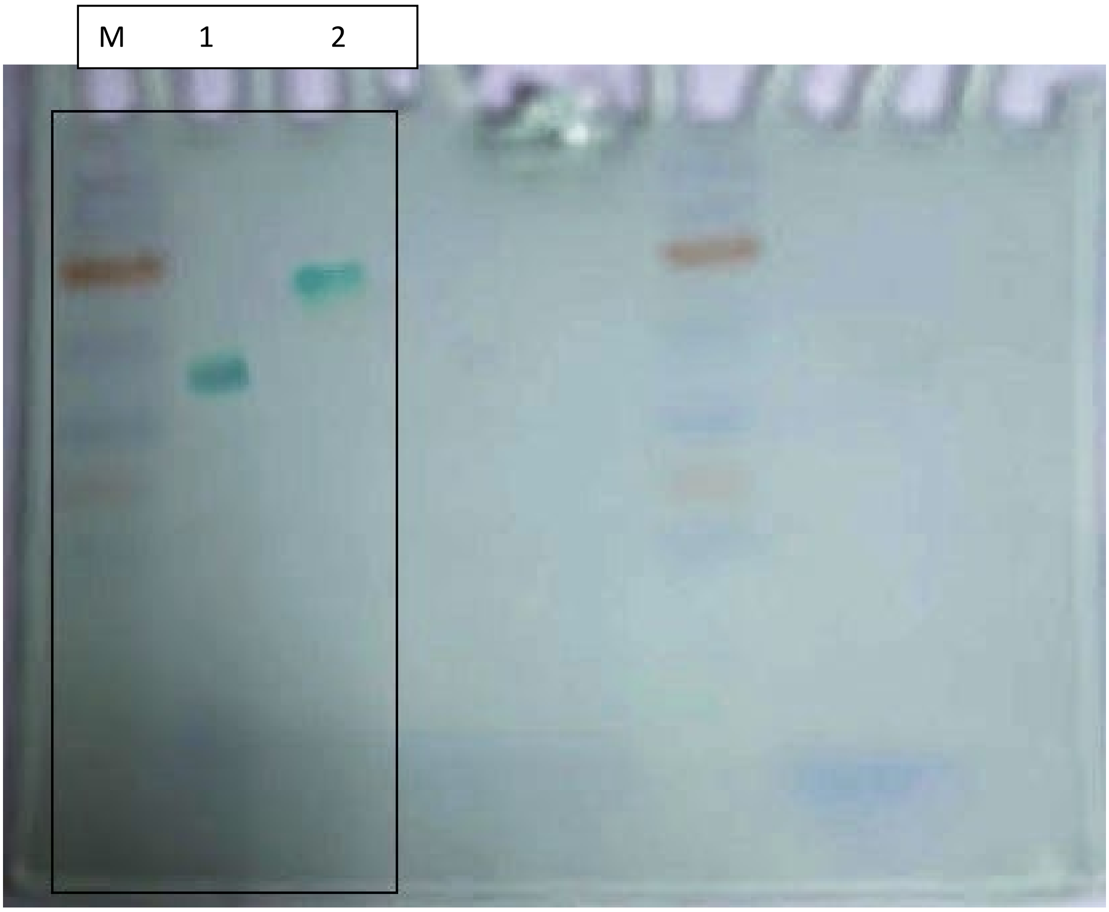

Figure S4: Venn diagram of the protein cluster (A), amino acid sequence (B) and scaffold view Laccases from *Tl*FLU1 and *Tp*FLU12 (C).

(A)

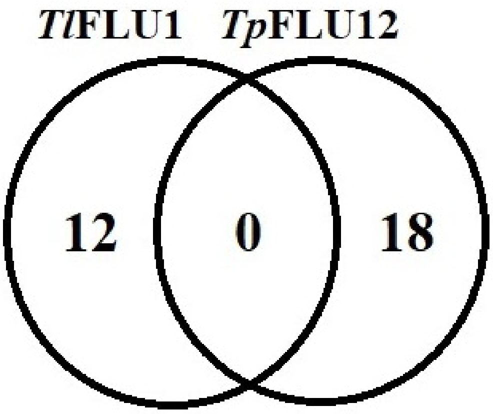

(B)

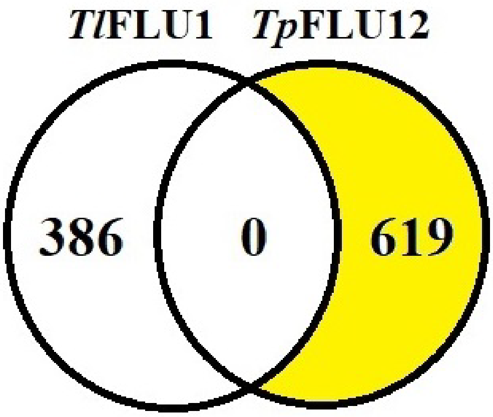

(C)

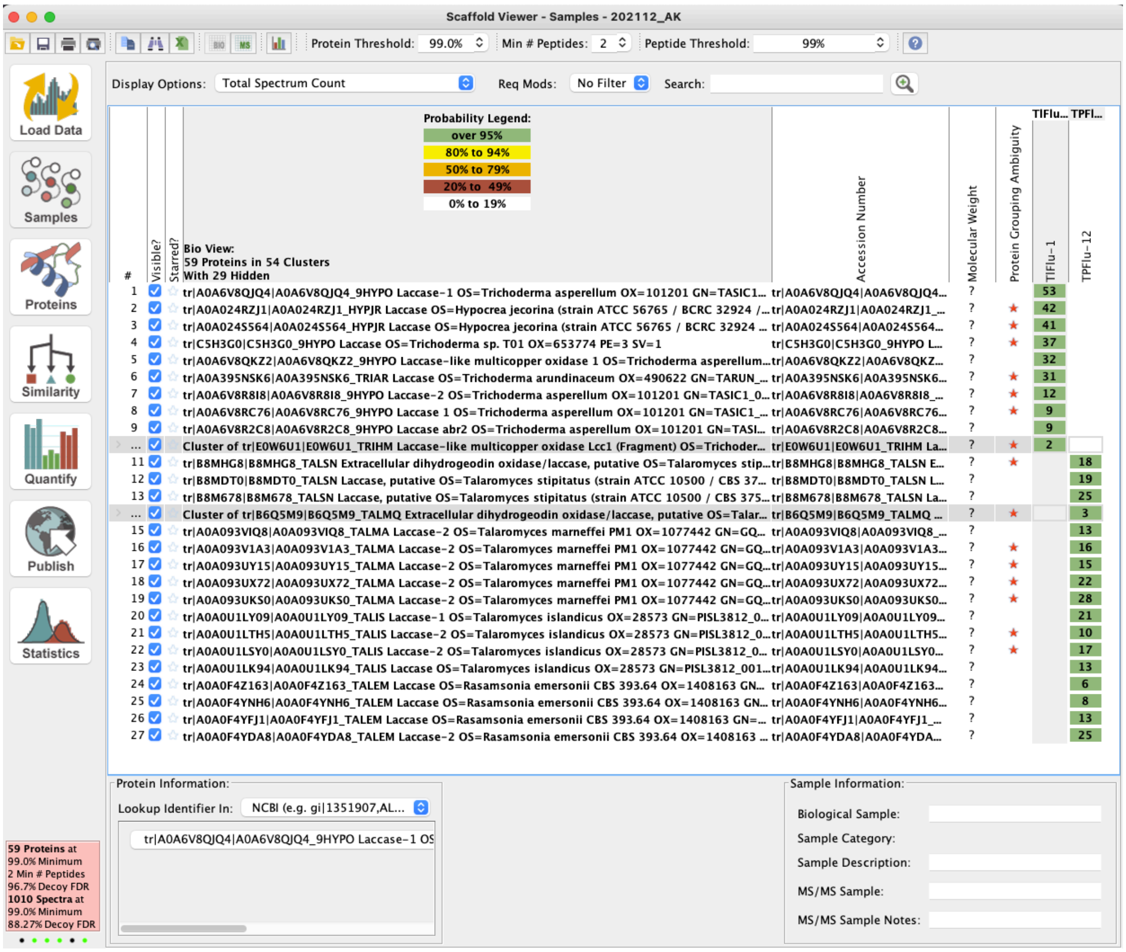

Figure S5. Neighbourhood-joining phylogenetic tree of 8 Laccases from the *Trichoderma* family and the reference RSB PDB sequence used as template for modelling the protein structure.

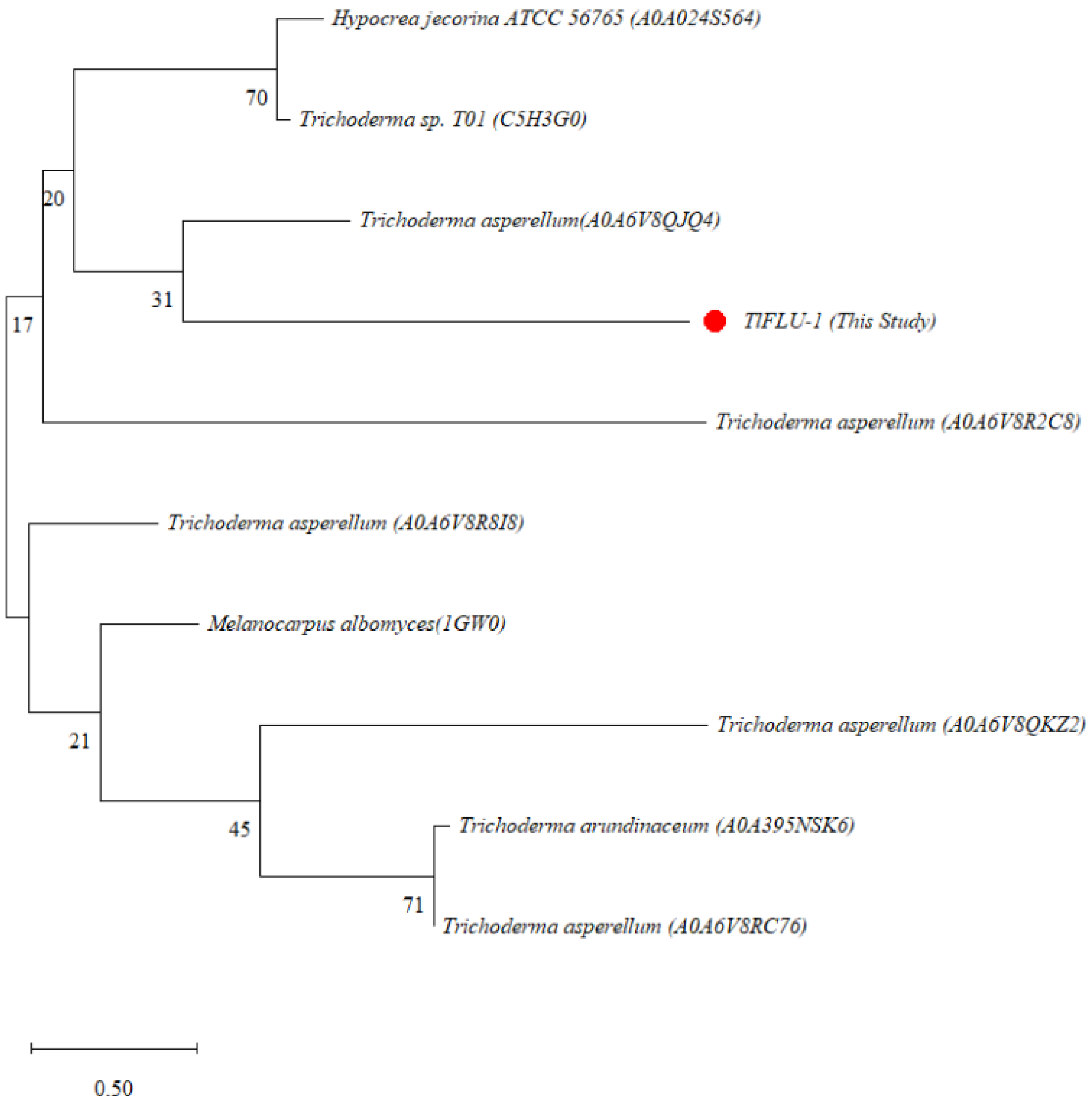

Figure S6. Neighbourhood-joining phylogenetic tree of 9 Laccases from the *Talaromyces* family and the reference RSB PDB sequence (4X4K) used as template for modelling the protein structure.

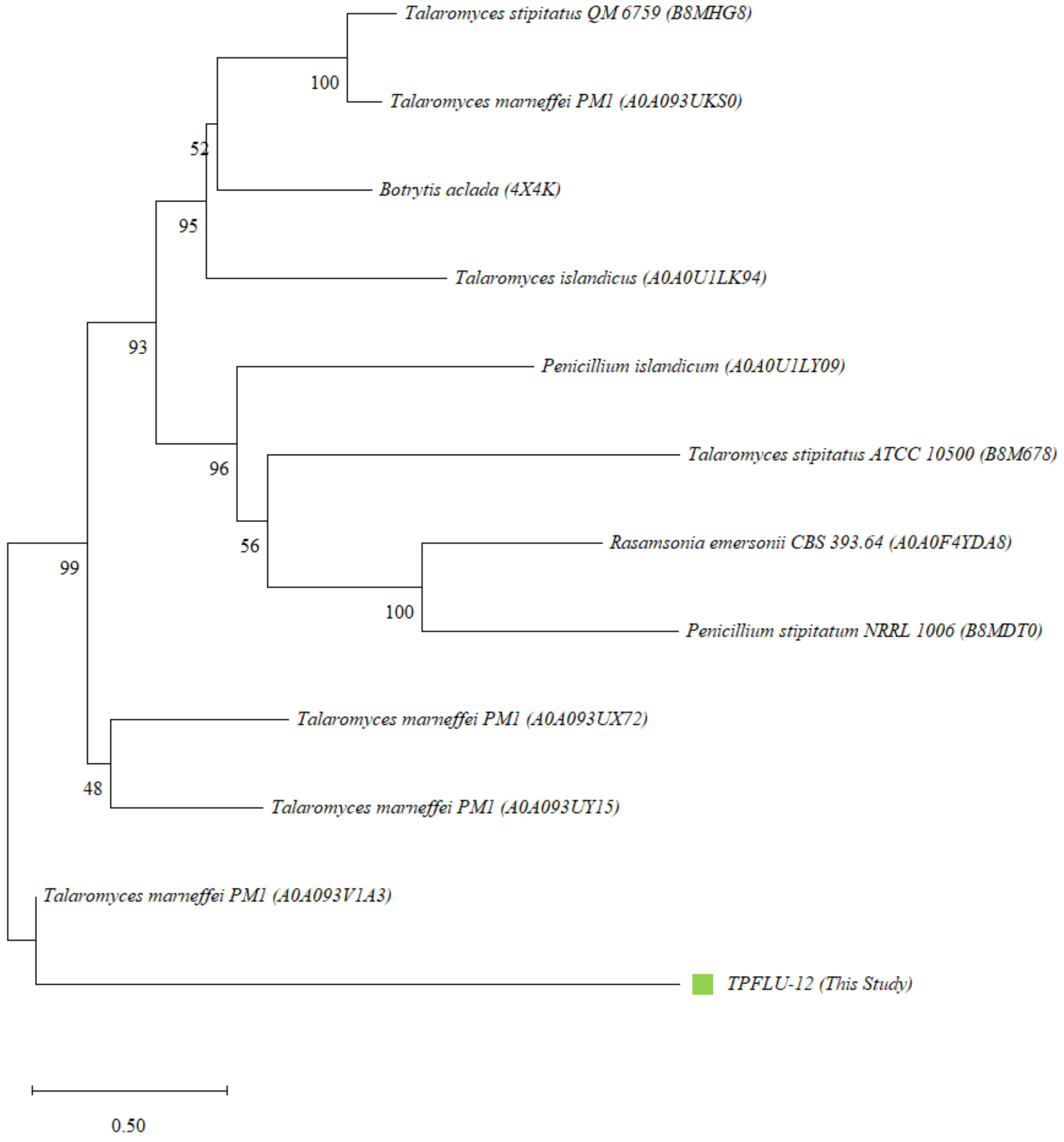

Figure S7. The homology sequence alignment of *Tl*FLU1 Laccase and IGW0 (*Melanocarpus albomyces*) template from the LOMETS threading programs of the I-TASSER server constructed with Jalview workbench.

a) Ident1 is the percentage sequence identity of the templates in the threading aligned region with the query sequence.
b) Ident2 is the percentage sequence identity of the whole template chains with query sequence.
c) Cov represents the coverage of the threading alignment and is equal to the number of aligned residues divided by the length of query protein.
d) Norm. Z-score is the normalized Z-score of the threading alignments. Alignment with a Normalized Z-score >1 mean a good alignment and vice versa.
e) The aligned sequence was selected in order of their ranking from the following threading programs: 1: FFAS-3D 2: SPARKS-X 3: HHSEARCH2 4: HHSEARCH I 5: Neff-PPAS 6: HHSEARCH 7: pGenTHREADER 8: wdPPAS 9: PROSPECT2 10: SP3.

In the consensus, '–' is a gap, '+' is an ambiguous position with no consensus while the most conserved residues are highlighted in a blue box.

Rank	PDB Hit	Iden1	Iden2	Cov	Norm. Z-score	Download Align.
1	1gw0A	0.13	0.24	0.81	1.78	Download

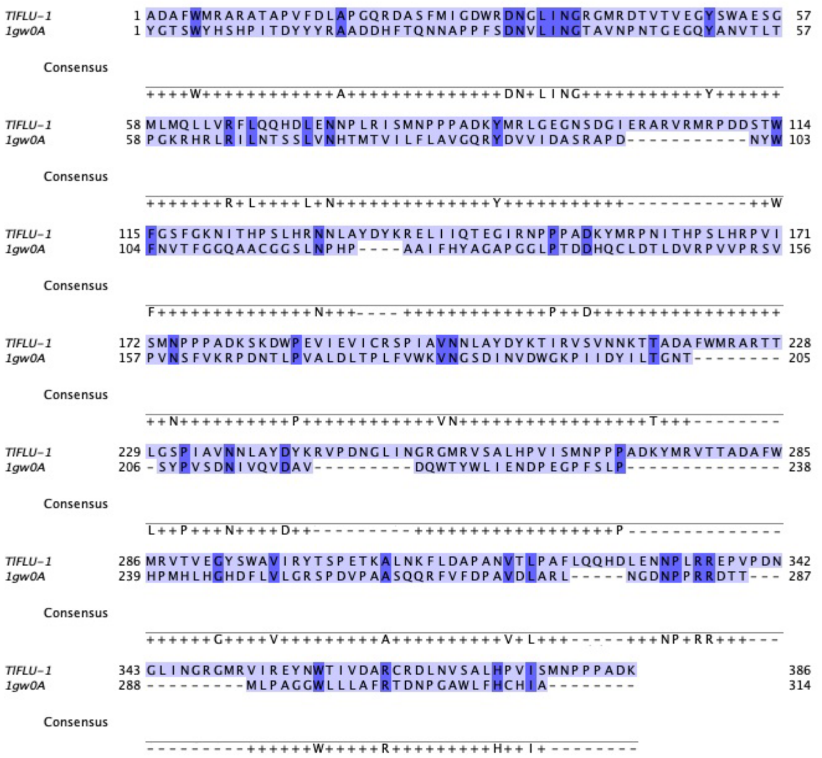

Figure S8. The homology sequence alignment of *Tp*FLU12 Laccase and 4x4kA (*Botrytis aclada*) template from the LOMETS threading programs of the I-TASSER server constructed with Jalview workbench.

a) Ident1 is the percentage sequence identity of the templates in the threading aligned region with the query sequence.
b) Ident2 is the percentage sequence identity of the whole template chains with query sequence.
c) Cov represents the coverage of the threading alignment and is equal to the number of aligned residues divided by the length of query protein.
d) Norm. Z-score is the normalized Z-score of the threading alignments. Alignment with a Normalized Z-score >1 mean a good alignment and vice versa.
e) The aligned sequence was selected in order of their ranking from the following threading programs: 1: FFAS-3D 2: SPARKS-X 3: HHSEARCH2 4: HHSEARCH I 5: Neff-PPAS 6: HHSEARCH 7: pGenTHREADER 8: wdPPAS 9: PROSPECT2 10: SP3.

In the consensus, '–' is a gap, while '+' is an ambiguous position with no consensus. The most conserved residues are highlighted.

Rank	PDB Hit	Iden1	Iden2	Cov	Norm. Z-score	Download Align.
1	4x4kA	0.16	0.18	0.73	5.85	Download

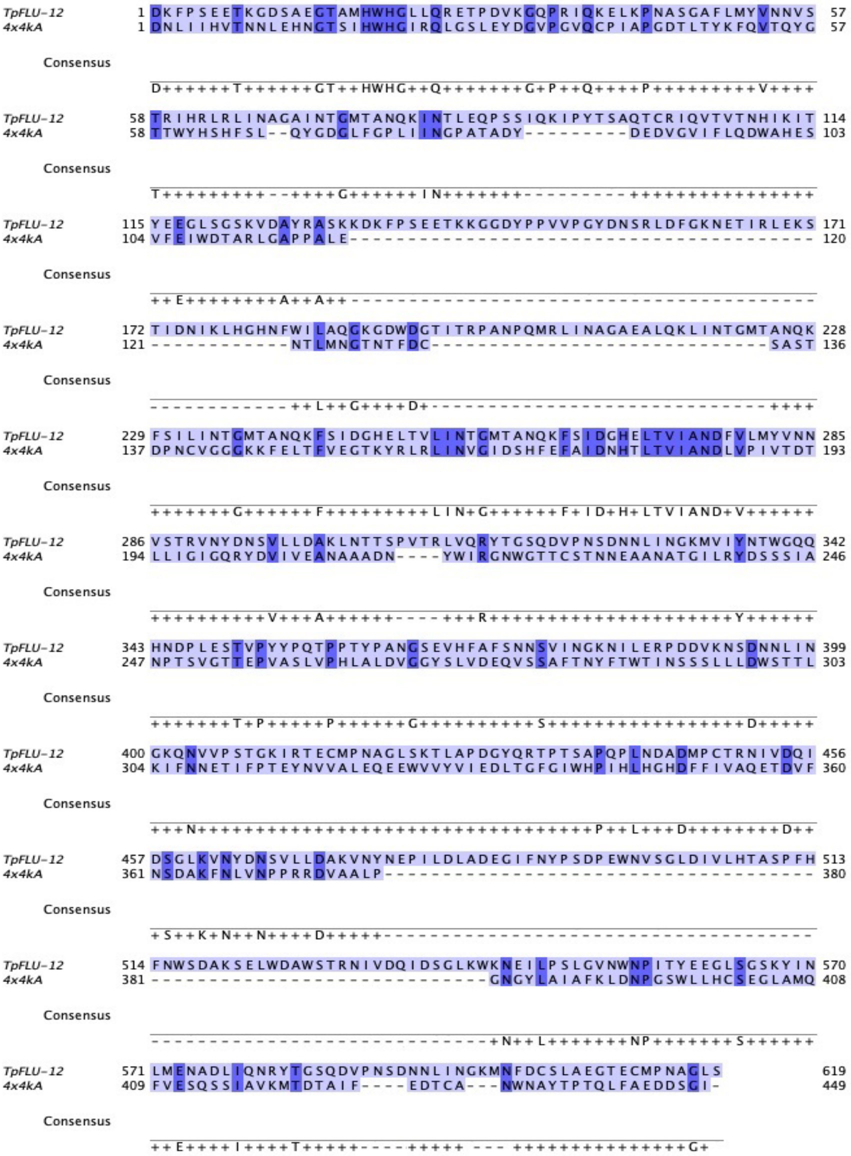

Table S1. The composition of the media used.

Media	Component	Amount (g/L)	Trace Elements	Amount (mg/L)
Basal salt media (BSM)	$(NH_4)_2SO_4$	2.4	Nitrilotriacetic acid	15
	K2HPO4	1.55	$CaCl_2.2H_2O$	15
	NaH2PO4.2H2O	0.85	$MnCl_2.2H_2O$	6
	NaCl	0.5	$FeSO_4.7H_2O$	1
	$MgSO_4.7H_2O$	0.26	$Co(NO_3).6H_2O$	1
			$ZnSO_4$	1
			$CuSO_4$	0.1
			H_3BO_3	0.1
			Na_2MoO_4	0.1
			$Al_2(SO_4)_3.H_2O$	0.1
Potato Dextrose Agar PDA	Potato Infusion (infusion from 200g potatoes)	4		
	D(+)-Glucose (=Dextrose)	20		
	Agar	15		
